# The State of the Art and Innovations in Active and Edible Coatings and Films for Functional Food Applications

**DOI:** 10.3390/polym17182472

**Published:** 2025-09-12

**Authors:** Sandra Mariño-Cortegoso, Antía Lestido-Cardama, Raquel Sendón, Ana Rodríguez Bernaldo de Quirós, Letricia Barbosa-Pereira

**Affiliations:** 1 FoodChemPack Research Group, Department of Analytical Chemistry, Nutrition and Food Science, Faculty of Pharmacy, University of Santiago de Compostela, 15782 Santiago de Compostela, Spain; antialestido@gmail.com (A.L.-C.); raquel.sendon@usc.es (R.S.); ana.rodriguez.bernaldo@usc.es (A.R.B.d.Q.); 2 Instituto de Materiales (iMATUS), University of Santiago de Compostela, 15782 Santiago de Compostela, Spain

**Keywords:** edible coatings, biobased materials, coating technology, control release, natural extracts, functional foods, safety and regulation

## Abstract

Edible coatings and films are gaining the attention of researchers, consumers, and the food industry as a sustainable alternative to conventional plastic packaging. This review provides an overview of recent advances in their development, with a particular focus on new natural sources of biomaterials (e.g., proteins and polysaccharides) and natural additives (antioxidants and antimicrobials). Special attention is given to high-technology preparation methods, including electrohydrodynamic atomization (EHDA), as well as controlled release systems for bioactive compounds designed to preserve foodstuffs and extended their shelf life. The application of edible coatings as carriers of nutrients (vitamins) and bioactives (probiotics and polyphenols) to improve the nutritional value and support the development of functional foods is also discussed. In addition, this review addresses safety considerations and regulatory aspects that are crucial for commercialization and consumer acceptance. Finally, key challenges are highlighted, including the improvement of mechanical and barrier properties, scalability of innovative technologies, consumer education, regulatory support, and the integration of circular economy principles, to encourage the adoption of these sustainable solutions.

## 1. Introduction

Biobased systems are emerging as effective alternatives to conventional plastic packaging [1]. Within this context, edible packaging arises as a promising option since it is recognized as safe, biodegradable, low-cost, and sustainable. Edible coatings and films are thin layers composed by food-grade ingredients applied in direct contact with the food. They can be consumed as part of the product while preserving its nutritional and sensory attributes [2]. Although both share similar biopolymeric matrices, their applications differ: films are preformed standalone layers that can be placed onto food surfaces, while coatings are generated directly on the product during application. This conceptual and functional difference is critical, as it determines their formulation, preparation methods, structural properties, and technological implications in food packaging. Edible coatings and films can be produced with biopolymers and active compounds extracted from agri-food residues. Such materials can improve food quality, extend shelf life, and reduce environmental waste [3,4]. The correct application of these biomaterials comprises the development of food contact materials with acceptable barrier and mechanical properties. In addition, the functional activity and nutritional value of edible coatings can be enhanced by incorporating additives, such as colours, flavours, antioxidants, antimicrobials, vitamins, and nutraceuticals, into their polymeric matrix [5]. To fulfil the desired function, an efficient encapsulation technique enables the compound of interest to be delivered to the target and express its functional activity after ingestion. Common production and encapsulation methods described in the literature include dipping, brushing, spraying, solvent casting, and extrusion [6].

Despite the benefits of using edible coatings and films to improve the quality, shelf life and safety, the commercial applications of many of these coatings are still rather limited [3]. Global regulations, industrial application, cost feasibility, consumer acceptance, and ensuring environmental sustainability are the biggest obstacles to the commercialization of these packaging systems. Consumer acceptance is mandatory for the successful development of food products and significantly influences the global food market. Increasing demand for healthy, safe, and sustainable foods has led to a greater acceptance of natural edible packaging. However, edible coatings and films sometimes provide undesirable flavours or potential toxicity to food products. Therefore, their acceptance relies not only on the functional properties, but also on other factors, such as film appearance, organoleptic properties, effective marketing, and the overall cost of the final food product [7]. While several reviews have summarized the fundamentals and general applications of edible coatings and films, important aspects remain underexplored. In particular, there is limited discussion on (i) the incorporation of new biopolymer sources and the exploitation of nanostructures and other advanced technologies to enhance mechanical and barrier properties; (ii) the design and verification of controlled release systems for bioactive compounds, which is still an emerging research area with high potential for food preservation; and (iii) the role of edible coatings as carriers of nutrients and bioactives to develop functional foods, an aspect that has not been sufficiently emphasized in earlier works.

This review addresses these gaps by providing an update perspective on recent materials and technologies, with special attention to controlled release strategies and their implications for food functionality. Furthermore, safety considerations, regulatory challenges, and their social and environmental impact are critically discussed to offer a comprehensive outlook on the opportunities and limitations of edible coatings and films.

## 2. Biopolymers for Edible Films and Coatings Development

The research development of active and edible coatings and films is currently focused on searching for suitable materials, such as polysaccharides and proteins, increasingly used as biopolymers [1]. As such, Table 1 displays current commercialised edible coatings and films. Additionally, fortification with bioactive compounds might enhance the capability to extend the shelf life of perishable food products, as well as to improve the physical limitations usually associated with these biopolymers [2]. Table 2 summarizes the state of the art in the development of biopolymer-based systems that incorporate bioactive or functional compounds through various technologies described in the following section. It outlines the types of biopolymers used, the nature of the active compounds, and the technologies employed for their integration. The table also details the specific food applications and highlights the resulting effects on both the material properties of the biopolymer system (such as mechanical strength, barrier properties, and biodegradability) and the preservation of food products (including antimicrobial activity, oxidation delay, and shelf life extension).

### 2.1. Proteins

A wide range of proteins from animal and plant sources can be employed as biopolymers. Gelatin is a denatured protein obtained from acid, alkali, or enzymatic collagen hydrolysis that shapes transparent, odourless, and tasteless films that demonstrated good gas barrier properties and high mechanical resistance and elasticity; although its high-water permeability limits its use in food packaging [8,9]. Gelatine is first dissolved in hot water to create a film-forming solution. The film is then produced using the casting technique, followed by drying. For instance, Moreno et al. (2020) [10] have developed an active, gelatine-based coating loaded with an ethanolic propolis extract that effectively extended raspberries’ shelf life by avoiding fungal growth.

Whey protein, constituted principally of β-lactoglobulins, *α*-lactalbumins, and bovine serum albumin, has gained relevance over recent years for being a by-product of cheese processing. It is tasteless, transparent, and has proved to be a controlled barrier against oxygen, but with high water permeability [11]. When the protein percentage is ∼90%, it is known as whey protein isolate, but when the content ranges 70–80% it is recognized as whey protein concentrate [12]. Whey-based films and coatings containing bioactive compounds have largely demonstrated its effect on shelf life extension by decreasing microbial growth and/or enhancing antioxidant properties. For example, Sajimon et al. (2023) [13] have observed a water resistance improvement on films loaded with oregano essential oil, while effectively extending the shelf life of grapes without affecting perceived sensorial quality (see Table 2).

However, it is important to use protein from plant-based resources for vegetarian diets and, if possible, agro-industrial by-products, aiming to reduce the environmental impacts that petroleum-based packaging and animal use lead to [14]. In this sense, soy protein has been used since the beginning of the century. It is derived from soybeans, which contain up to 44% protein, primarily classified as globulins, with β-conglycinin and glycinin being the major fractions [15,16,17]. Soy protein is water-soluble and commonly requires the addition of plasticizers or other biopolymers to enhance its mechanical and barrier properties in film-forming solutions [18]. For instance, Mostafa et al. (2023) [19] reported that the incorporation of cellulose nanocrystals into soy protein isolate films significantly improved their mechanical strength. Additionally, the introduction of date palm leaf extract conferred antioxidant properties. However, the liquid form of the extract reduced tensile strength, while the powder form preserved the mechanical performance of the original blend. In another study, Yousuf et al. (2020) [20] developed a coating solution comprising soy protein isolate and lemon extract, which was applied to fresh-cut melon. The coating effectively retained vitamin C, reduced microbial growth, and improved key physicochemical properties, thereby extending the shelf life of the fruit.

Zein protein, the main storage protein and a residue in corn starch production, has been investigated over the last few years. Zein is an amphiphilic protein, composed by more than 50% of hydrophobic amino acids, which depending on their structural setup, can be exploited to formulate a hydrophobic surface for food packaging applications. It is considered that the rate of solvent evaporation is a key step to control surface hydrophobicity. As such, electrohydrodynamic processing can lead to the formation of superhydrophobic surfaces due to high-speed solvent evaporation, while samples prepared by solvent casting induced self-assembly with resulting hydrophilicity behaviour due to conformational transitions from α-helix to β-sheet [21]. It has a good film-forming capacity, high tensile strength, and low water and oxygen permeability, and it can be used to encapsulate bioactive compounds, which can improve barrier properties through the formation of non-covalent bounds, mainly H-bonds and hydrophobic interactions [22,23]. Xia et al. (2023) [24] have developed a hydrophobic film based on peppermint oil–zein nanofibers further coated by sprayed methyltriethoxysilane. They studied the effect of solvents (aqueous acetic acid and ethanolic solutions) on a zein protein secondary structure. As result, they observed that β-chain was predominant in acetic acid solutions, while α-helix was the major structure found using an ethanolic solution. Furthermore, they observed that hydrophobic behaviour was further enhanced by peppermint oil loading, which increased antioxidant and antimicrobial properties, successfully prolonging the shelf life of coated pork.

Silk fibroin has gained significant attention in recent years as a promising material for edible coatings and films. It is the main structural protein of silk, typically accounting for 65–85% of its composition [25]. Due to its high β-sheet content, which can be further increased through water annealing treatments, silk fibroin exhibits excellent gas barrier properties. This was demonstrated by Marelli et al. (2016) [26] in the preservation of strawberries, where increasing the β-sheet content up to 58% significantly enhanced the fruit’s shelf-life. Building on this approach, Jaramillo-Quinceno and Restrepo-Osorio (2020) [27] improved the performance of silk fibroin coatings using the same annealing methodology. Notably, they successfully extracted silk fibroin from fibrous silk waste for the first time, and applied it to fruit coatings. Their study also included an analysis of metal contaminants, indicating a low-to-moderate risk for human consumption, thus supporting its potential for safe food applications. Reflecting the growing industrial interest, Mori^®^ has already commercialized an edible silk fibroin-based coating in the USA, designed for various food types, primarily fruits and vegetables, to effectively extend their shelf life (see Table 1).

### 2.2. Lipids

#### 2.2.1. Resins

Shellac is a resin produced by the *Kerria lacca* insect composed primarily of long-chain aliphatic hydroxy acids and sesquiterpenoid acid, which make it insoluble in water. Its unique physicochemical properties, including low vapour permeability, high gloss, and good adhesion, have led to its use across various fields, such as pharmaceuticals, food, or archeology [28,29,30,31]. In the food industry, shellac is approved as a food additive under the code E904 [32,33]. In food packaging applications, shellac is widely employed as a coating, particularly for fruits and confectionery, to enhance moisture resistance, appearance, and shelf life. Due to its versatility, it is also explored as a delivery system of bioactive compounds through the development of nanofibers, nanoparticles, microparticles, and microcapsules [34,35]. Its mechanical and functional properties can be further improved through polymer blending or chemical modification, expanding its potential in active packaging systems.

#### 2.2.2. Waxes

Waxes are among the most historically used materials in edible coating applications [15]. Although they are not classified as biopolymers—due to the absence of a repeating monomeric structure—their functionality in food packaging is well recognized. They are often directly applied as coatings or as components in composite films with proteins or polysaccharides [36,37]. Chemically, waxes consist predominantly of long-chain aliphatic esters, fatty acids, and alcohols, which confer strong hydrophobicity. This property significantly enhances their barrier performance, particularly by reducing water vapour permeability [38]. As a result, wax-based coatings effectively limit moisture loss and reduce weight loss in coated food products [39]. Additionally, the incorporation of bioactive compounds—such as polyphenols or plant-derived extracts—further enhances their functional properties. These bioactive-enriched systems can impart antioxidant and antimicrobial activity, contributing to improved food quality and extended shelf life [40,41]. Commonly used waxes include beeswax, carnauba, and candelilla waxes. More recently, rice bran wax obtained from rice processing by-products was more recently employed, due to its circular economy approach. As such, Abhirami et al. (2020) [42] employed a 10% *w*/*v* rice bran wax emulsion to successfully extend tomato shelf life up to 27 days, compared to 18 days for the control.

### 2.3. Polysaccharides

On the other hand, polysaccharides attract attention in food packaging for their gelling properties. Chitosan results from the deacetylation of chitin, a biopolymer found in crustaceous exoskeletons. It has a relevant impact in the development of non-plastic alternatives in food packaging due to its biodegradability, non-toxicity, biocompatibility, antimicrobial properties, and film-forming ability, in addition to its demonstrated elasticity [43,44]. As such, Davoodi et al. (2020) [45] have employed chitosan as a functional additive to mucilage-based films to enhance biopolymer performance and provide antibacterial activity to extend the shelf life of cherries and apples. Moreover, many authors have developed active chitosan films or coatings with phenolic compounds, phenol-rich extracts, or essential oils to enhance antioxidant properties and extend food shelf life [46,47].

Nevertheless, as has occurred with protein biopolymers, research recently has been conducted on plant-based alternatives. In this sense, starch and cellulose from different sources have been widely employed for decades [48,49]. Starch is the most abundant polysaccharide, composed of amylose (20–30%) and amylopectin (70–80%) in different proportions depending on the origin. It is mainly obtained from potato, corn, wheat, rice, barley, cassava, etc. [50,51]. In starch-based films, additives are required to improve their inherently poor mechanical and barrier properties. To address the brittleness caused by starch’s crystalline structure, thermoplastic starch (TPS) is produced, typically through extrusion and by blending with plasticizers, such as glycerol. Kumar et al. (2024) [52] have developed starch-based active coatings incorporating clove essential oil and glycerol as a plasticizer. The authors observed wide differences between starch of various origins (mango kernels, corn, and litchi seed), with litchi seed starch films being the most effective at preserving mandarins.

Cellulose and its derivatives, such as carboxymethyl cellulose (CMC), hydroxypropyl methylcellulose (HPMC), or nanocellulose, have been extensively investigated and used in blends and laminates. Cellulose is formed by the polymerization of D-glucose through a β-1,4 glycosidic bond. Both starch and cellulose blends are mostly employed in industrial applications. However, cellulose’s water insolubility and crystallinity make cellulose-based alternatives more suitable for packaging development [53]. CMC is a cost-effective anionic cellulose derivative produced through the incorporation of the –CH_2_COOH group into the cellulose chain. Its water solubility causes a superior film-forming capacity than that of cellulose. It provides good oxygen barrier properties, higher flexibility, and high-water-vapour permeability films and coatings [54]. HPMC is a non-ionic derivative formed by the inclusion of –OCH_3_ and –CH_2_CHOHCH_3_ groups, which has gained attention over the years because of its water solubility and improved mechanical properties compared to CMC, its gel-forming capacity, and its ability to encapsulate bioactive compounds [55,56]. As such, Iqbal et al. (2024) [57] have developed a nanoemulsion to be applied as an edible coating made of HPMC, beeswax, Tween 80 as a surfactant, glycerol as a plasticizer, and thyme, cinnamon, and peppermint oil as functional additives, effectively extending the shelf life of sweet cherries. On the other hand, nanocellulose, typically classified into cellulose nanocrystals (CNCs) and nanofibers (SNFs), has attracted growing interest regarding the development of composites and coatings, particularly for its ability to reinforce the mechanical and barrier properties of biopolymers [58,59,60]. Moreover, the formation of H-bonds and electrostatic interactions not only enhance matrix integrity but also promote the encapsulation and controlled release of bioactive compounds [61,62]. For instance, Wardak et al. (2024) [60] developed an edible coating that significantly increased the shelf life of tomatoes using various concentrations of CNFs to reinforce starch-based films, which improved their mechanical, thermal, and barrier properties. CNCs and CNFs are generally extracted from plant biomass through acid hydrolysis or alkaline treatments [63]. However, more environmentally friendly approaches, such as enzymatic hydrolysis and bacterial production, have recently gained attention [64,65]. Among these, bacterial cellulose (BC) stands out due to its high purity and uniform nanofibrillar structure. These characteristics have enabled BC to find a wide range of food industry applications, not only in packaging but also as a food additive or ingredient, where it serves as an emulsifier, stabilizer, and thickener [66]. In packaging, BC’s high crystallinity, mechanical strength, and barrier properties make it an excellent reinforcement agent. For example, Li et al. (2023) [67] used BC as a nanofiller in biopolymer films, significantly enhancing crystallinity, thermal stability, and barrier performance, while also improving the shelf life of fresh-cut apples through superior freshness retention properties.

Arabinoxylans are a type of hemicellulose found in the cell walls of various cereal grains, such as rice, wheat, or corn. They are composed of xylose units forming the backbone, with arabinose side chains. Due to their excellent film-forming ability, arabinoxylans can produce transparent and flexible films suitable for food packaging applications. Moreover, arabinoxylans exhibit natural antioxidant properties, attributed to phenolic compounds, such as ferulic acid, that remain bound to their structure during their extraction. Their biopolymer functional performance can be enhanced by incorporating bioactive compounds, nanocomposites, or blending with other polymers [68,69,70].

Gums are diverse group of polysaccharides. Depending on their origin, they include marine gums (e.g., alginate, carrageenan, and agar), plant gums (e.g., gum arabic and gum Tragacanth), seed gums or galactomannans (e.g., guar gum), mucilages (e.g., psyllium gum), and microbial gums (e.g., pullulan, kefiran, xanthan gum, and levan) [71,72].

Algae are a rich source of polysaccharides commonly used in food packaging development. Alginate, composed of guluronic acid and mannuronic acid units, is a water-soluble biopolymer that has displayed moderate oxygen and low water barrier properties. Its film fragility and mechanical properties can be improved with plasticizers. It has gained importance in the field of food packaging owing to its ability to prepare hydrogels and to retain water, thus controlling the moisture levels in the packaged food, and the encapsulation and controlled-release capacities of its bioactive compounds [73,74]. Thus, Das et al. (2020) [75] prepared an alginate-based nanoemulsion loaded with *Citrus sinensis* essential oil to provide antibacterial activity to coatings to extend the shelf life of tomatoes. Carrageenan is another polysaccharide derived from seaweed; specifically, it is obtained from the Rhodophyceae family. It is water-soluble and used for its film-forming capacity, barrier, and antibacterial properties. Blends with other polysaccharides improve its thermal stability and poor mechanical and barrier properties [76,77]. Agar is a polysaccharide derived from marine red algae that consists of D-galactopyranose and 3,6-anhydro-L-galactopyranose, and is soluble in hot water. Despite it being used for its good film-forming capacities, its poor water vapour barrier properties, thermal stability, mechanical properties, brittleness, and high hydrophilia limit its application in food packaging. To overcome these drawbacks, agar is used in food coatings or films with other polymers, nanomaterials, or plasticizers [78,79].

Some bacterial polysaccharides, such as xanthan gum, have been widely used in the food industry for decades. In contrast, others like kefiran and levan have more recently gained attention due to their bioactive properties and film-forming abilities, making them promising candidates for edible and active food packaging.

Kefiran is an exopolysaccharide composed of glucose and galactose units, predominantly by *Lactobacillus kefiranofaciens*, the principal bacteria in kefir grains [80]. Kefiran exhibits antioxidant and antimicrobial activity, and forms transparent films with good barrier properties, enhancing its potential for food packaging applications. As with many other polysaccharides, its inherent brittleness often needs the incorporation of plasticizers or other biopolymers to improve flexibility and mechanical performance [81,82]. Additionally, the integration of bioactive compounds can modify the structural and functional properties of kefiran-based films. For instance, Hasheminya and Dehghannya (2024) [83] developed active kefiran–gelatin films incorporating a *Zhumeria majdae* essential oil nanoemulsion. The films exhibited reduced water vapour permeability and tensile strength, while showing significant improvements in elongation at break and light-blocking capacity. The nanoemulsion also enhanced the antioxidant, antimicrobial, and antifungal properties of the film, which was effective in preserving sponge cake over 16 days of storage [83].

Levan, another bacterial exopolysaccharide, is produced by species such as *Bacillus subtilis* and *Zymomonas mobilis*. It consists of β-(2→6)-linked fructose units and shares similar functional properties with kefiran, including film-forming capacity and antioxidant and antimicrobial activities [84]. However, like kefiran, levan-based films tend to be brittle, a limitation that can be addressed by blending with other biopolymers to enhance flexibility and functional performance. As such, Wang et al. (2022) [85] incorporated levan into chitosan-based films, improving their mechanical strength, UV light absorption, and surface hydrophobicity, while reducing swelling and water vapour permeability. The resulting films exhibited good thermal stability and were effective in preserving fresh pork, as indicated by a reduction in total volatile basic nitrogen [85]. Furthermore, levan-based systems can be functionalized by incorporating additional bioactive agents. In a study by Gan et al. (2022) [86], a levan–pullulan–chitosan composite coating enriched with ε-polylysine was applied to strawberries. The coating demonstrated significant improvements in microbial control, moisture retention, and oxidative stability, effectively extending the shelf life of the fruit.

On the other hand, fungal exopolysaccharides are also gaining interest as biopolymers. Among them, pullulan is the one that is most employed for food packaging applications. It is composed of maltotriose units bound through α-1,6 glycosidic bonds. It is a water-soluble biopolymer with poor water barrier properties but is an excellent barrier against oxygen. It displays moderate mechanical properties and thermal stability. The incorporation of bioactive compounds can improve the physical–chemical properties of pullulan-based coatings [87,88]. Therefore, An et al. (2023) [89] demonstrated that pullulan coatings enriched with *Auricularia auricular* extracts not only exhibited enhanced mechanical properties but also provided antioxidant and antimicrobial capacity. These improvements extended the shelf life of fresh-cut potato by reducing weight loss and limiting browning index, among other quality-preserving effects.

Scleroglucan, schizophyllan, and lentinan are other examples of fungus-derived exopolysaccharides. Sclerogucan is produced by *Sclerotium rolfsii*, schizophyllan by *Schizophyllum commune*, and lentinan by *Lentinula edodes* (shiitaki mushroom) [90,91,92]. While all three share a β-(1→3) backbone with β-(1→6) branching, they exhibit variations in their branching degree, solution behaviour, and biofunctional attributes.

These polysaccharides typically adopt a triple-helical conformation in aqueous environments, which contributes to their functional stability. Scleroglucan is valued for its excellent rheological properties, exhibiting shear-thinning behaviour, thermal resistance, and pH tolerance (maintaining viscosity up to pH 13 before decreasing) [93,94]. Schizophyllan also shows high thermal and salt stability and has demonstrated antioxidant activity, with primary applications in biomedicine [95]. For example, Hamedi et al. (2021) [96] developed a schizophyllan/bacterial cellulose-based hydrogel incorporating ZnO nanoparticles for wound healing purposes. However, no studies have yet reported its use in food packaging [96].

All three polysaccharides are currently utilized in cosmetics and biomedical formulations, but scientific research on their application in edible coatings or films remains scarce. In food systems, scleroglucan and schizophyllan are mainly used as stabilizers, thickeners, and gelling agents, particularly in Japan [97]. On the other hand, lentinan has demonstrated several beneficial effects, highlighting its antioxidant, antimicrobial, and lipid-lowering properties, as well as resistance to gastric digestion and high bioavailability, which makes it a promising candidate for edible coating applications [92,98,99]. A recent example is provided by Cui et al. (2023) [100] who developed a lentinan-based film for beef preservation. To overcome the brittleness of the polysaccharide matrix, the researchers incorporated soy protein nanoparticles loaded with *Litsea cubeba* essential oil. The resulting composite film demonstrated significantly improved mechanical properties, gas barrier performance, and thermal stability. Moreover, it exhibited strong antioxidant and antibacterial activities, effectively delaying lipid oxidation on the beef surface for up to seven days, thereby extending its shelf life [100].

Other known gums, such as xanthan gum, mucilage, gellan gum, dextran, or locust bean gum, are also currently being studied and their ability as biopolymers to incorporate bioactive compounds to extend food shelf life is being proven. Thus, Aayush et al. (2024) [101] have developed a nanoemulsion with xanthan gum, with Tween 80 as an emulsifier and betel leaf extract, to effectively extend the shelf life of tomatoes by 6 days by improving several physical–chemical properties.

Tamarind polysaccharide is an underexploited biopolymer for food packaging applications that has recently emerged as a biopolymer, considering tamarind seeds form a large proportion of the fruit and are already being exploited by the pharmaceutical industry for drug delivery purposes. It is mainly composed of xyloglucan, based on units of xylose, galactose, and glucose. Contrary to most of the previously described biopolymers, tamarind polysaccharide possesses good mechanical and moderate barrier properties [101,102,103,104].

Finally, among polysaccharides, pectin is being considered as a biopolymer due to being easily recuperated from industrial fruit by-products, mainly apples and citrus peels. Depending on its purification process, it can possess antioxidant capacity due to the covalently bound phenolic compounds, but can also be included to functionalize pectin-based films [105,106]. Its water retention capacity makes it a material of interest in moisture control to enhance the shelf life of food. Its combination with plasticizers improves its mechanical properties, especially regarding flexibility aspects [107].

The abridgments that all biomaterials have shown regarding mechanical and barrier properties can be improved by modifying the polymeric structure using plasticizers (e.g., glycerol, sorbitol, or polyethylene glycol) [108], crosslinking agents (e.g., citric acid or CaCl_2_) [109,110], nanomaterials, or grafting bioactive compounds [111,112]. Furthermore, the inclusion of bioactive compounds, such as polyphenols, functionalizes films and coatings by providing antioxidant, antifungal, or antimicrobial properties. This functionalization primarily involves the use of phenolic compounds (e.g., ferulic acid or resveratrol) [113], natural extracts (e.g., rosemary or lemon extracts) [114], essential oils (e.g., cinnamon or oregano essential oils) [115], aldehydes (e.g., linalool or citral) [116], or organic acids (e.g., acetic or sorbic acid) [117].

## 3. Technologies for Edible Coatings and Film Development and Applications in Food

**Figure 1:**
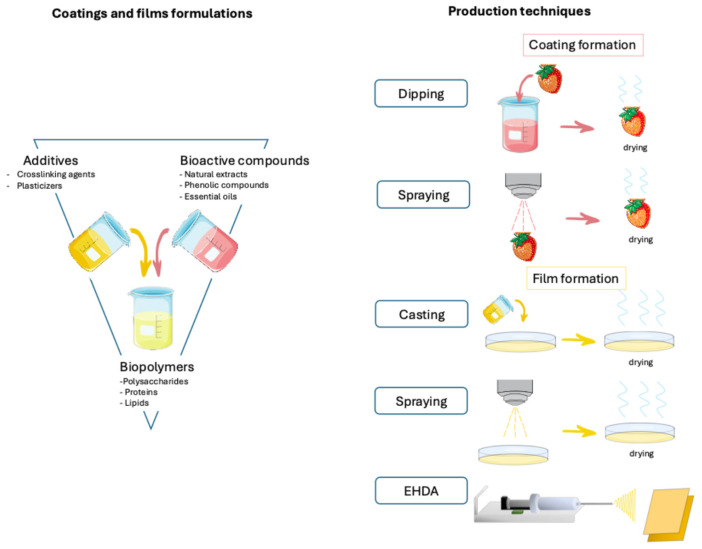
Formulation and application methods of edible biopolymer-based coatings and films for food applications.

The development of active packaging to extend food shelf life has driven significant advances in techniques for producing and applying films or coatings on food products. These technologies enable the incorporation of bioactive compounds, particularly natural extracts rich in phenolic compounds or isolated phenolics, which provide antioxidant and antimicrobial properties. Recent years have seen a growing trend in optimizing these methods to enhance both the functional performance of the coatings and the preservation of food quality. Figure 1 illustrates the formulation of edible coatings and films using biopolymers, additives, and bioactive compounds such as antimicrobial agents, plasticizers, and essential oils. It also shows common production techniques used for their application or formation, including dipping, spraying, casting, and electrohydrodynamic atomization (EHDA). Coatings are typically applied directly onto the food surface, while films are formed separately and later applied. Each method involves a drying step to stabilize the coating or film. All the methodologies described, both for coating application and for film formation, are found in Table 2, which summarises the state of the art in the development of biopolymer-based systems that incorporate bioactive or functional compounds through the technologies described in the current section.

### 3.1. Films Fabrication Methods

Melt extrusion is a thermomechanical processing technique that involves melting a biopolymer/blend, which is subsequently passed through a die to form continuous films. The resulting films are then solidified by cooling down. It is a technique widely employed in industry for petroleum-based or biobased material processing due to its high production, versatility, and low-cost. Currently, it is also investigated for its application in edible biopolymers. As such, Cheng et al. (2022) employed blown extrusion to produce ε-polylysine hydrochloride-loaded starch/gelatin edible antimicrobial films [118]. Additionally, Huntrakul et al. (2020) have developed acetylated cassava starch and pea protein isolate films, successfully improving starch processability in blown extrusion and barrier properties [119].

Injection moulding is a manufacturing process broadly used in conventional plastics production. This method is emerging for employment in the production of edible and active films for food packaging applications. It involves a mould to obtain the desired shape of a directly injected molten material, which is then solidified. Recently, Plasencia et al. (2025) [120] have developed compostable and thermoplastic zein-based films, demonstrating the feasibility of injection moulding processing in producing films with valuable physicochemical properties.

Casting is a low-cost technique widely employed to develop active films, especially at a lab-scale level. It consists of solubilizing the materials for further solvent evaporation, incorporating directly the bioactive compounds and other additives into the polymeric matrix [121,122,123]. Alterations in the biopolymer structure to improve its biopolymer performance and enabling the controlled release of bioactive compounds are highly employed in the development of active coatings and films. In this sense, the addition of crosslinking agents, including molecules, such as CaCl_2_, citric acid, or cinnamaldehyde is a commonly used technique. Whitehead et al. [124] demonstrated that adding genipin as a crosslinking agent has led to an impact on morphological matrix characteristics and, consequently, on the delivery of ascorbic acid. Phenolic compounds can also act as crosslinking agents because of their binding affinity to the polymeric matrix, which directly depends on the number of hydroxyl groups [125,126]. Thus, phenolic compounds can improve mechanical and barrier properties, while performing antioxidant action.

In recent years, research into encapsulation technologies has significantly increased, focusing on protecting bioactive compounds from environmental degradation caused by factors such as humidity, UV light, and heat, while also enhancing their controlled release.

The use of nanoemulsions to ameliorate the solubility of bioactive compounds and entrap them into the biopolymers to provide a controlled release has increased in the last 10 years [127]. Nanoemulsions are more homogeneous and provide higher encapsulation efficiency compared to microemulsions. Their lower particle size (20–200 nm) is related to a large surface volume that allows slow diffusion rates [128]. The efficacy of an active coating loaded with a nanoemulsion was verified by Y. Xiong et al. [129] since the preservation and coating stability was enhanced by the inclusion of oregano essential oil and resveratrol in a nanoemulsion system. Different high-energy and low-energy methods are required to develop nanoemulsions, with the former highlighted for its extended use of high-pressure techniques and ultrasonication [130]. Due to the thermodynamic instability of these formulations, surfactants are employed to reduce the interfacial tension, avoid coalescence and flocculation phenomena, and to reach the desired small particle size [131].

Finally, electrohydrodynamic atomization (EHDA) has arisen as an encouraging technique in active packaging development due to its capacity to transform biopolymer solutions into nanostructures, generating films or coatings. This technique employs an electric field to produce electrospun nanofibers or electrosprayed nanoparticles, ranging in size from the micrometric to the nanometric. Both structures’ properties can be modulated by modifying parameters, such as the material concentration, needle opening, and distance-to-collector or voltage [132,133]. Electrospun nanofibers and electrosprayed nanoparticles have a high porosity and surface area-to-total volume ratio that allow the loading and controlled release of the encapsulated bioactive compounds, which aids in improving water and oxygen barrier properties [134]. Zein electrospun nanofibers developed by M.A. Moreno et al. [135] reached encapsulation efficacies of ~90% and increased the controlled release of phenol-rich extracts by applying glutaraldehyde as a crosslinker agent. One of the main advantages of EHDA is its versatile use since it can process a wide range of biopolymers, as is also the case with non-electrospinnable materials, using recent advances in its setups (e.g., coaxial or trilayer) [136].

Multilayer formulations have been shown to improve the physicochemical properties of films and coatings. Among the various techniques for producing multilayer films, the electrohydrodynamic atomization (EHDA) of multilayer films has proved to enhance the performance characteristics of the films, boosting mechanical strength and flexibility, while also improving their functionalization with bioactive compounds [137,138].

### 3.2. Coating Application Methods

Coating formulations unloaded or loaded with bioactive compounds can be applied as coatings using various methods, with dipping being the most common and cost-effective technique. In this process, the food product is immersed in the solution, followed by the evaporation of solvents, leaving a protective coating on the surface [139,140,141,142].

Spraying, another widely used coating technique, offers the advantage of precise control over key factors in the coating process, including system conditions, operational parameters, and the structural characteristics of the polymer. This method produces uniform layers with well-controlled thicknesses, ensuring the consistency and quality of the final coating [52,143]. Thus, Zhong et al. (2014) [144] have observed that spraying and electrostatic spraying led to thinner coating deposition on Mozzarella cheese among all biopolymers tested (alginate, chitosan, and soy protein).

Spreading or brushing techniques consist of the direct application of the coating with a brush. They are commonly used for small fruits and vegetables. However, the drawbacks of these techniques are related to the non-controlled coating thickness and the limitations in terms of large-scale applications [145,146]. Hence, Rajaei Lak et al. (2024) [147] found that, of the studied methods, brushing is the least effective, just below the dipping method [147].

Vacuum impregnation is a useful technique for applying coatings to porous fruits and vegetables, but is not suitable for delicate fruits. It is a non-thermal technique which removes the air and moisture from the food matrix, followed by coating infusion. It allows a thicker deposition, higher concentration, and controlled deposition compared to the dipping technique [148,149]. Vacuum impregnation can also be employed to enhance the colour, texture, flavour, and aroma of porous foods [150]. When combined with osmotic dehydration, vacuum impregnation can increase mass transfer efficiency while minimizing structural alterations and reducing process duration [151]. The technique is inherently complex and strongly influenced by multiple intrinsic and extrinsic variables, making process optimization essential for achieving optimal efficiency and consistent product quality. Senturk Parreidt et al. (2018) [152] evaluated the effect of dipping and vacuum impregnation on melon coated with an alginate-based formulation. The authors observed that vacuum impregnation was more effective in terms of firmness, weight, and colour preservation than the dipping method.

The panning method is well-established in the food industry and, in particular, the pharmaceutical industry, due to its efficiency in processing large food quantities. It involves placing food products in a large rotating pan while the coating solution is evenly sprayed onto their surfaces as the pan continuously spins. Thus, it is especially useful for spherical foodstuffs, such as nuts. It ensures a homogeneous distribution of coating whose thickness can be controlled by the pan’s rotation speed. After coating application, food is commonly subjected to a drying process [153,154,155].

The enrobing method is another methodology commonly employed in the food industry for applying coatings, particularly for confectionery products. This process involves dipping or passing food through a curtain or bed of coating material or formulation, such as molten chocolate, to achieve a uniform and continuous coating. As such, enrobing is commonly employed for products like chocolate-covered nuts [144,156]. Rajaei Lak et al. (2024) [147] evaluated the effect of applying a zein-based coating enriched with *Heracleum persicum* essential oil onto cheese using different methods. The results demonstrated that the enrobing method was the most effective in preserving the cheese’s physicochemical properties, notably by reducing microbial counts, minimizing pH fluctuations, and limiting moisture loss.

Fluidized-bed processing method consists of solid particles suspension in a stream of air or gas, enabling thin and uniform coating layer application on small and dry food products. It is followed by a drying process [157,158]. Moreover, fluidized-bed-processing is a useful method for encapsulation purposes, enhancing product functionality. It is a high-cost technique compared to those previously reported, although it is greatly employed by the food industry [159,160].

Cold plasma treatment is an innovative technique used in food packaging applications to enhance the properties of edible coatings. It involves generating plasma by applying an electric field to a neutral gas under atmospheric pressure, exposing materials to low-temperature plasma. Various approaches, including laser treatment, ozonation, UV radiation, or gamma rays can be employed to induce physicochemical changes on biopolymers. Plasma-based methods are utilized to improve the functional characteristics of biopolymers for the intended applications, such as adhesion or barrier properties [161,162]. For instance, Akhavan-Mahdavi et al. (2023) [163] successfully extended pistachio shelf life using a cold plasma and chitosan coating, thus reducing mould and yeast growth and aflatoxin concentration, without significantly altering the other physicochemical characteristics or sensory perception.

Ultrasonic spray coating is a next-generation spraying technique that enables multilayer coating through layer-by-layer deposition, promoting bond interactions between layers and addressing issues such as cross-contamination and time-consuming processes. Ultrasonic spray coating produces uniform droplets with a consistent size and distribution, ensuring a high-quality coating. The technique allows for the incorporation of various additives and materials, facilitating the encapsulation of bioactive compounds and the creation of multifunctional coatings [164,165]. Additionally, UA operates at low pressure, requiring less energy for atomization, making it an efficient and versatile method for advanced food coating applications [166]. Some studies have explored the advantages of its use for conventional polymers or its application as a technique for food products, ingredients, or bioactive compound processing, but it is currently an underexploited technology in terms of the development of edible and active films or coatings [167,168].

All the described techniques have shown encouraging potential in the production of edible and active coatings or films. However, more studies should be performed to overcome the drawbacks regarding scalability and cost-effectiveness for large-scale packaging applications.

**Table 2:** Biopolymer-based systems incorporating bioactive compounds, production technologies, food applications, and their effects on material properties and food preservation.

Biopolymers	Bioactive/Functional Compounds	Technology	Application	Effect	Ref.
*Proteins*					
Gelatin	Grape seed extract	Coating dispersion	Tilapia fillets (vacuum impregnation)	Protective effects against metabolic changes. ↓ changes contents the main metabolites. Inhibited the formation of harmful substances or undesirable compounds with off-odours. ↓ pH changes, ↓ TVB-N, and ↓ K-value variations	[169]
Gelatin	Peppermint and chamomile EOs	Electrospinning	NA	↑ WCA, ↑ antioxidant properties (>chamomile EO), ↑ antibacterial activity (>peppermint EO), and not cytotoxic	[134]
Zein–gelatin	Tea polyphenol	Casting (multilayer stacking)	Kiwifruit, avocado and banana (packaged)	Fruits: ↓ WL, ↓ ΔE, ↑ TPC, and ↓ microorganism growth	[170]
Irradiated starch–gelatin	Lime juice	Casting	Chicken (packaged)	Films: ↑ mechanical properties and ↓ microorganism growth Chicken: ↓ lipid oxidation and ↑ SL (12 days)	[171]
Gelatin	Propolis extract	Casting (extract encapsulation in zein nanocapsules–antisolvent precipitation)	Raspberries (dipping)	Films: ↑ flexibility, ↑ film colour, and ↑ antifungal activity Raspberries: ↑ SL (11 days)	[10]
Gelatin	Tomato by-product hydrolysate	Casting	Pork loin (dipping)	Pork: ↑ WL, ↑ ΔE, ≃ pH, ≃ water activity, ↓ lipid oxidation, and ↑ antioxidant activity	[172]
Gelatin	Cinnamaldehyde	Casting (Cinnamaldehyde-sulfobutyl ether-β-cyclodextrin inclusion complex)	Grass carp fillets (dipping)	Films: ↑ flexibility, ↑ opacity, and ↓ microorganism growth Carp fillets: ↓ protein degradation	[173]
Gelatin–chitosan	Curcumin and cinnamon oil	Casting (pickering emulsion of cinnamon oil with oxidized CNF)	Pork meat (covered)	Film: ↓ L*, ↑ ΔE, ↑ roughness, ↑ TS, ↓ EB, ↓ WCA at 0 min, ↑ WCA at 10 min, ↓WVP, ↑ antioxidant capacity, and ↑ antibacterial properties Pork meat: ↓ TVB-N, ↑ SL, and freshness indicator (ΔE)	[174]
Gelatin–pectin	Lemongrass EO	Casting	Chicken breast (packaged)	Films: ↓ L*, ↓ WS, ↓ mechanical properties, ↑ thermal stability, ↓ microorganism growth, and ↑ antioxidant activity Chicken: ↓ WL and ≃ pH	[175]
Whey	Natamycin or/and α-tocopherol nanoemulsion (o/w)	Casting	NA	Films: ↓ TS and elastic modulus, ↑ opacity, ↑ ΔE, ↑ WVP, ↓ microorganism growth (natamycin), and ↑ antioxidant activity (α-tocopherol)	[176]
Sweet whey–starch	Chlorogenic acid	Casting	Bananas (dipping)	Films: ↑ UV barrier, ↑swelling, ↑ toughness, ↓ microorganism growth, and ↑ antioxidant activity Bananas: ↑ antioxidant activity and ↓ browning	[177]
WPI	A511 bacteriophage	Casting	Cheese (dipping)	Films: ≃ phage viability (2 weeks) and ↓ microorganism growth Cheese: ↑ colour, ↑ hardness, ↑ springiness, ≃ adhesiveness, ≃ cohesiveness, ≃ gumminess, and ≃ chewiness	[178]
Tomato seed mucilage–whey	Shallot EO	Casting	Fish fillet (dipping)	Fish fillet: ↑ L*, ↓ WL, ↓ hardness, ↓ chewiness, ↓ springiness, ↓ cohesiveness, ↓ gumminess, ↓ pH, ↓ TBA, ↓ TVB-N, ↓ ΔE, and ↓ microorganism growth	[179]
Nanochitosan–WPI	Summer savoury EO	Casting	Rainbow trout fillets (dipping and packaged in pouches)	Films and fish: ↓ microorganism growth	[180]
Pectin–WPC	*Lactobacillus helveticus*	Casting	Acid-Curd Cheese (dipping)	Cheese: ≃ colour, ≃ moisture, and ↓ microorganism growth	[181]
WPI	Transglutaminase	Casting (microemulsion)	Fresh-cut apple (dipping)	Films: ↑ mechanical properties and ↑ water resistance Apple: ↓ WL, ↓ browning index, ↓ PPO activity, ↓ CAT activity, ↓ H_2_O_2_ production, and ↓ MDA accumulation	[182]
WPC	Green tea extract	Casting	Fresh Cheese (packaged)	↑ antioxidant activity Cheese: ↓ lipid oxidation and ↑ ΔE cheese	[183]
WP	NA	Coating dispersion	Peanuts	↓ lipid oxidation and ↑ SL (3 months)	[155]
WP	NA	Coating dispersion	Chocolates (pan coating)	WPI–sucrose provided chocolates with the most gloss (even after 5 months). Sucrose crystallization contributed to coating gloss	[154]
WPI	Bergamot oil	Casting (nanoemulsion with nanocellulose)	NA	↑ mechanical properties, ↓ WVP, ↓ L*, ↑ opacity, ↑ homogeneous structure, and ↑ antioxidant activity	[184]
WPC	Oregano EO	Casting	Grapes (packaged)	Films: ↓ mechanical properties, ↓ microorganism growth, ↑ antioxidant activity, ↓ WS, and ↓ light transmittance Grapes: ↓ ΔE grapes, ≃ weight, ≃ pH, ≃ acidity, ≃ Brix value	[13]
Zein	Methyl ferulate	Coaxial electrospinning	Sea Bass (wappred)	Films: ↑ TS, ↑ fibre membrane, ↓ crystallinity, ↑ thermal stability, ↓ WCA, ↓ microorganism growth, irregular network structure, smooth surface, ↑ membrane hydrophilicity, and sustained release Sea bass: ≃ pH and ≃ TVB-N content	[185]
Zein–PVA–chitosan	Anthocyanin extract	Layer-by-layer casting	Shrimp (covered)	Films: ↑ mechanical properties, ↑ flexibility, ↓ WVP, and UV protection Shrimp: ↑ antioxidant activity and colorimetric response to TVB-N (↑ ΔE)	[186]
Zein	Garlic EO	Casting (nanoemulsion)	Vannamei prawn (dipping)	Prawn: ↓ lipid oxidation, ↓ TVB-N, ↓H_2_S production, ↓ microorganism growth, ↑ antioxidant activity, ↑ SL, and ↑ acceptability	[187]
Zein-chitosan	Thymol; thyme, cinnamon and oregano EOs	Casting (micellar particles)	Strawberries (dipping)	Micelles: ↓ particle size and ↓ ζ potential Strawberries: ↓ moisture loss, ↓ microorganism growth, and ↑ SL	[188]
Zein	*Heracleum persicum* EO	Coating dispersion	Cheese (Brushing, dipping, spraying, enrobing)	Cheese: Hardness preserved, ↓ microorganism growth, ↓ moisture loss, ↓ lipid oxidation, ↓ pH increase, ↑ overall acceptability over storage, ↑ SL, and ≃ L*. Enrobing > spraying > brushing > dipping	[147]
Zein–beeswax	Nisin	Coating dispersion	Nectarines and apples (dipping)	Nectarines and apples: ↓ WL, ↑ firmness, ↑ antibacterial activity, and ≃ mould and yeast growth	[189]
Zein	2-hydroxypropyl-ß-cyclodextrin	Casting	Strawberries (dipping)	Films: ↓ zein aggregation, ↑ zein solubility, ↓ light transmission, ↑ hydrophilia, ↑ WVP, ↓ moisture content, ↑ UV-light blocking properties, ↑ TS, ↓ strain at break, ↓ YM, ↓ WVP, and ↓ antioxidant properties Strawberries: ↓ WL, ↓ microorganism growth, and ↑ SL	[139]
Zein	Dill leaf extract and dill leaf EO	Casting (EO encapsulation in β-cyclodextrin)	Carp fillets (wrapped)	Films: ↑ antibacterial properties, ↓ moisture content, ↑ thermal stability, ↑ L*, ↑ surface roughness, ↑ TS (with extract, EO free and encapsulated), ↓ TS (with extract and EO, free or encapsulated, combined), ↑ EB (with extract, EO free), and ≃ EB (with EO encapsulated; extract and EO, free or encapsulated, combined) Fillets: ↓ microorganism growth, ↓ lipid oxidation, ↓ pH increase, ↓ TVB-N, ↑ desirable aroma, ↑ overall acceptability over storage time, and ↑ SL	[190]
Starch–zein	Sorghum bran extract	Casting	NA	↑ TS, ↑ EB, ↓ moisture, ↓ WS, ↑ opacity, ↑ antioxidant activity, ↓ microorganism growth, and ↑WVTR	[191]
Zein-chitosan	NA	Electrospinning (zein) Casting (zein and chitosan) (bilayer films)	Apple slices (wrapped)	Films: ↑ moisture permeability, ↑ EB and ↓ TS (with ↑ PEG), and ↑ antioxidant activity Apples: ↑ anti-browning ability and ↑ WL	[192]
Zein	Thyme EO	Electrospinning	Strawberries (packed in a PET container with the active film attached to its lids)	Nanofibers: linear morphology, smooth surface and bead-free structure, and physical encapsulation process Strawberries: ↓ microorganism growth, ↓ biochemical changes, ↑ SL, and ≃ antioxidant properties for 15 days	[193]
Zein–CNC	Curcumin	Core–shell microparticles (2:1 zein-CNCs) Solvent evaporation (curcumin loaded zein microparticles) Antisolvent precipitation	NA	↓ particle size and ↑ stability with ↑ curcumin, ↓ particle size at ↑ pH, ↓ bioaccessibility with ↑ CNCs, ↑ degradation under UV light, and CNCs ↑ thermal stability	[194]
Zein	TiO_2_ nanotube arrays (TNTAs)	Casting (bilayer films, TNTAs-zein)	NA	↑ mechanical strength, ≃ flexibility and ↑ water resistance (compared to zein), and ↑ antimicrobial activity	[195]
Zein–chitosan	Eugenol or/and curcumin	Casting	Blueberries (dipping)	Films: shear-thinning behaviour, ↑ TS, ↑ EB, ↓ WVP, ↓ oxygen and CO_2_ permeability, ↓ surface roughness, ↓ L*, and ↑ ΔE Blueberries: ↑ UV and pH stability, ↓ microorganism growth, ↑ antioxidant properties, ↓ WL, and ↑ hardness	[196]
Zein–chitosan–dialdehyde CMC	Cinnamaldehyde	Casting (cinnamaldehyde–zein antisolvent precipitation)	Strawberries (packed)	Films: ↑ mechanical strength, ↓ WVP, ↓ oxygen permeability, ↑ UV-light blocking properties, ↑ hydrophobicity, ↓ WS, ↓ moisture, ↓ L*, and ↓ ΔE Strawberries: ↓ WL, ↓ microorganism growth, ↑ antioxidant properties, and ↑ SL	[197]
Zein	NA	Injection moulding	NA	↑ thermal stability, ↓ water uptake along weeks, ↑ EB, ↓ elongation (↑ urea), and ↓ toughness (↑ urea). Clearer colour and ↑ hydrophilic properties (↑ urea). Plasticizers ↓ glass transition temperature. 80% degradation after 6 weeks	[120]
Sodium caseinate	NA	Blown extrusion (co-rotating twin-screw extruder)	NA	↓ Young’s modulus (↑ glycerol % and ↓ relative humidity), ↑ EB (glycerol ≥ 25% and ↑ RH), and ↑ WVP (↑ glycerol)	[198]
Pea protein isolate	NA	Injection moulding	NA	↓ film transparency. ↑ TS, ↑ Young’s modulus, and ↓ EB (70% pea protein). ↑ water uptake. 30–40% glycerol needed for ↑ processability	[199]
*Polysaccharides*					
Chitosan	NA	Coating dispersion (substitution of wheat flour by chitosan solution in frying batter)	Fish sticks (enrobing)	↑ L*, ↓ whiteness, ↓ Hue angle, ↓ fat uptake, ↓ TVB-N, ↓ PO value, ↓ lipid oxidation, ↓ hardness, ↓ crispness, ↓ gumminess, ↓ Warner–Bratzler shear force, and ↓ toughness	[200]
Chitosan	Kojic acid and clove EO	Coating dispersion	White shrimp (dipping)	↓ microorganism growth, ↓ TVB-N, ↓ pH, ↓ ΔE, ↑ sensory scores, and ↓ WL	[201]
Chitosan–gelatin–catechol-modified chitosan	AgNP	Casting (catechol–chitosan synthesis)	NA	↓ WS, ↑ surface roughness, ↑ TS, ↓ EB, ↓ WVP, and ↑ antibacterial properties	[202]
Chitosan	Gallic acid	Casting (gallic acid encapsulation in ZnO NP)	NA	↑ TS, ↑ EB, ↓ WVP, ↓ oxygen permeability, ↓ swelling, ↓ WS, ↑ UV-barrier properties, ↑ antibacterial properties, and ↑ antioxidant properties	[203]
*Salvia macrosiphon* seed mucilage–chitosan	NA	Casting	Cherries and apples (dipping)	Films: ↑ thermal stability, ↑ TS, ↓ EB, ↓ WVP, ↓ WS, ↓ moisture content, ↑ UV-barrier properties, ↑ transparency, and ↑ roughness Fruits: ↑ antibacterial properties and ↑ SL	[45]
Chitosan–hydrolysed orange peel (pectin-rich)	NA	Casting	NA (overall migration assay in Tenax^®^)	↑ elastic modulus, ≃ TS, ↓ EB, ↑ oxygen barrier properties, ↓ WVP, ↑ thermal properties, ↓ moisture content, ↑ UV-barrier properties, ↑ WS, ↑ transparency, ↑ antioxidant capacity, ↑ antibacterial properties, and fast biodegradability (62.9% WL after 26 days)	[204]
Collagen–chitosan	Gallic acid and ε-polylysine	Casting (grafting of gallic acid onto chitosan backbone)	Pork (wrapped)	Films: ↑ UV-barrier properties, ↓ L*, ↑ ΔE, ↑ WS, ↑ WVP, ↓ TS, ↓ EB, ≃ thermal stability, ↑ antioxidant capacity, and ↑ antibacterial properties Pork: ↓ TVB-N, ↓ lipid oxidation, and ↑ SL (5 days)	[47]
Chitosan–guar gum	Watermelon rind extract	Casting	Fresh-cut bananas (packaged)	Films: ↑ TS, ↑ EB, ↓ WVP, ↓ oxygen permeability, ↑ thermal stability, ↓ L*, ↓ moisture content, ↓ WS, ↑ antioxidant capacity, ↑ antibacterial properties Bananas: ↓ WL, ↑ firmness, ↓ TSS, ↑ sensory quality, and ↑ SL	[205]
Chitosan–chitin	Eggplant anthocyanins	Casting (chitin nanofibers development)	Pork (film fixed on the top of the box)	Nanofibers: ↓ NP ζ potential Films: ↑ compactness, ↑ TS, ↑ EB, ↑ thermal stability, ↑ hydrophobicity, ↓ L*, ↑ ΔE, ↑ WVP, ↑ oxygen permeability, ↓ moisture content, ↓ WS, ↑ UV barrier properties, ↑ antioxidant capacity, ↑ antibacterial properties, pH sensitivity, ammonia sensitivity, acid sensitivity, and colour changes easily detected by the naked-eye	[206]
Chitosan	Luteolin	Casting (luteolin encapsulation in o/w nanoemulsion)	NA (specific migration assay in EtOH 95%)	Nanoemulsion: ↓ ζ potential, PDI ∼ 0.2 Films: ↑surface homogeneity, ↓ L*, ↑ ΔE, ↑ compactness, ↓ WVP, ↓ oxygen permeability ↑ TS, ↑ EB, ↑ antioxidant capacity, ↑ antibacterial properties, and slow controlled release rate	[207]
Carboxymethyl chitosan	Pomegranate peel extract	Coating dispersion	Grass carp fillets (vacuum impregnation; packaged in sterile bags)	↓ drip loss, ↓ texture softening, ↑ sensory scores, ↓ lipid oxidation, ↓ TVB-N, ↓ K-value, ↓ bioamine accumulation, ↓ microorganism growth, and ↑ SL (3 days)	[46]
Chitosan	NA	Coating dispersion	Pumpkin (dipping and vacuum impregnation)	Vacuum impregnation: ↑ thickness, ↑ incorporation, ↑ homogeneity, ↓ water content loss, ↑ pH variations, ↑ acidity changes, ↑ ΔE, and ↑ firmness	[208]
Chitosan	NA	Coating dispersion	Pistachio (dipping and cold plasma treatment)	preserved hardness and colour indices, ↓ PO, ↓ microorganisms growth, ↓ aflatoxins, and ↑ overall acceptance (1.5% chitosan coating and 120 s of cold plasma treatment, most effective)	[163]
Chitosan	Pomegranate extract	Hydrogel pad	NA	↑ swelling ratio over time and ↑ pH, porous hydrogel (cross-linking), ↑ TPC, ↑ antioxidant capacity, ↑ antibacterial properties, and water absorption	[209]
Cassava starch–pea protein	NA	Blown extrusion	Soybean and olive oil (sachets)	Pea protein: stabilized films during blown-extrusion, ↓ flexibility, ↓ non-homogeneity AS-PI blend matrices. ↑ TS, ↓ WS, ↓ light-transmission, ↑ crystallinity, ↑ WCA, ↓ WVP, ↓ OP, ↓ humidity-induced shrinkage, and ↑ thermal stability	[119]
Cassava starch–chitosan–gelatin	NA	Coating dispersion	Guavas	↓ WL, ↓ titratable acidity, ↓ vitamin C, ↑ soluble solids, ↓ ripening index, ↓ microorganism growth, and ↑ SL (9 days)	[210]
Starch–gelatin	ε-polylysine	Blown extrusion	Bread (wrapped)	Films: ↓ complex viscosity, ↓ storage modulus, ↑ gelatinization degree, ↑ WCA, ↓ WVP, ↑ WS, ↓ TS, ↑ EB, and ↑ antimicrobial effect Bread: ↓ microorganism growth and ↑ SL	[118]
Potato starch	Carvacrol	Casting	Paipa cheese (brushing)	Films: ↓ WS, ↓ TS, ↓ YM, ≃ WVP, ≃ moisture content, ≃ transparency, ≃ swelling behaviour, and ↑ antioxidant properties Cheese: ↑ brightness, ≃ water activity, ≃ moisture content, ≃ colour attributes, ↓ microorganism growth, ↑ hardness, ↑ gumminess, ↑ springiness, and ↑ chewiness	[146]
Sweet potato starch	Cumin EO	Coating gel	Pears (dipping)	↓ rot lesion on infected pear caused by *Alternaria alternata*, ↓ changes in fruit colour, ↓ firmness changes, ↓ chlorophyll degradation, suppressed the onset of climacteric rise in respiration, ↓WL, ≃ stomata densities, ↓ microorganism growth, and ↑ sensory quality	[140]
Jackfruit starch	Pomegranate peel extract	Casting	Grapes (dipping)	Film: ↑ TPC, ↑ antioxidant properties, ↑ thermal stability, ≃ WS, ↑ TS (↓ concentrations), ↑ WVP, ↑ oxygen permeability Grapes: ↓ WL, ↑ firmness, and ↑ L*	[211]
Starch	NA	Coating dispersion	Strawberries (dipping, then packed in PET containers)	↓ ripening, ↓ firmness reduction, ↓ WL, ↓ total soluble solid reduction, ↓ ascorbic acid loss, ↓ microbial load, and ↓ redness reduction	[142]
Mango kernel, corn, and litchi seed starch	Clove EO	Casting (Ultrasonication)	*Khasi* mandarins (spraying)	Formulations: ↑ consistency (mango < litchi < corn starch), ≃ particle size, ↓ ζ potential (litchi < mango < corn starch), and ↓ PDI (corn < litchi < mango starch) Films: ↑ TS, ↓ transparency, ↓ WS (litchi < corn starch), ↑ antioxidant capacity, and ↓ microorganism growth Mandarin: ↓ firmness reduction, ↓ WL, ↑ TPC ↑ TFC, ↑ antioxidant activity, ↓ respiration rate, and ↓ TSS Litchi seed starch–CEO—ultrasonication, the most effective	[52]
Buckwheat starch–xanthan gum	Lemongrass EO	Casting (Ultrasonication)	Plum (dipping)	Films: ↓ moisture content, ↓ WS, ↑ L*, ↑ thermal properties (compared to commercial starch), ↑ TS, ↓ EB, ↓WVP, ↑ WCA, ↑ antioxidant capacity, and ↑ antimicrobial activity Plum: ↓ ripening, ↓ WL, ↓ TSS, ↓ pH increase, and ↓ shrinkage	[212]
Potato starch	Sodium benzoate	Coating emulsion	“Fino” lemons (dipping)	↓ WL, ↓ gas exchange, ≃ firmness, ≃ pH, ≃ Hue angle variation, ≃ titratable acidity, and ↓ disease severity and incidence (lemons were inoculated with *Penicillum digitatum*)	[141]
Corn starch	Fumaric acid	Coating dispersion	Silver pomfret fish steaks (dipping; individually packed in nylon-EVOH-PE pouches)	Bacteriostatic effect (↑ antibacterial activity), ↓ H_2_S production, ↓ pH increase, ↓ TVVB-N increase, ↓ lipid oxidation (↓ TBA, ↓ PV increase) ↑ overall acceptability reduction, and ↑ SL (9 days)	[213]
Starch–CNF	Citric acid (crosslinking agent)	Casting	Tomatoes (dipping)	Films: ↑ TS, ↑ EB, ↑ thermal stability, ↓ WVP, ↓ swelling degree, and ↓ transparency Tomatoes: ↓ WL, ↓ firmness reduction, ↓ TSS loss, ↓ pH increase, and ≃ colour	[60]
CNF	Carbon dots	Casting	Tangerine, strawberry (dipping; migration assays in H_2_O, 10, 50 and 95% EtOH)	Films: ↑ UV blocking properties, ≃ transparency, ≃ mechanical properties, ↑ WVP, ↓ oxygen permeability, ↑ WCA, ↑ antioxidant properties, ↑ antimicrobial activity, negligible cytotoxicity, and ↑ CDs release in more hydrophilic simulants Fruits: inhibited fungal growth and ↑ SL (>10 days for tangerine, >2 days for strawberries)	[214]
Alginate–CNC	Thyme and/or clove EO	Casting (pickering emulsion)	Guava (dipping)	Films: ↓ viscosity, ↑ particle size, ↑ opacity, ↓ WS, ↓ WVP ↑ antioxidant properties, and ↑ antifungal activity Guavas: ↓ WL, ↓ hue angle decrease, ↓ pH increase, ↑ vit C retention, ↓ firmness reduction, ↓ titratable acidity reduction, and ↓ TSS increase	[61]
HPMC	Rutin	Casting (liposomes)	NA	↓ YI, ↑ ΔE, ↓ moisture, ↓ TS, ↑ EB, and ≃ WVP (compared to edible coating with free rutin)	[215]
HPMC–beeswax	Thyme, cinnamon and peppermint oil	NA Nanoemulsion	Sweet cherries (dipping)	↓ total suspended solids, ↓ titratable acidity, ≃ colour, ↓ WL, ↓ respiration rate, ≃ firmness, ↓ TPC reduction, ↑ sensory quality, and ↑ antimicrobial activity	[57]
HPMC	NA	Coating dispersion	“Golden Reinders” and “Granny Smith” apples (dipping)	↓ firmness reduction, ≃ colour, ↓ superficial scald, ↓ antifungal growth, ↓ ACS activity, ↓ ethylene production, ≃ starch index, ≃ soluble solids content, ≃ total titratable acidity, ↓ VOCs (↓ terpenoids, ↓ esters, ↓ aldehydes) ↓ consumer acceptance, ↓ α-farnesene levels and its oxidation products, and ↑ ethanol accumulation Most effective in “Golden Reinders” apples	[216]
HPMC–chitosan–alginate	Nisin	Casting (bilayer)	Chestnut (dipping)	Films: ↑ WVP, ↑ oxygen permeability, ↑ WS, ↓ transparency, ↑ thermal stability, ↓ TS, and ↓ EB Chestnut: ↓ respiratory intensity, ↓ WL, and ↓ decay rate	[217]
CMC	Polysaccharide and phenolic compounds from spent coffee grounds	Coating dispersion	Goldenberries (spraying)	↓ WL, ↓ gas permeability (O_2_, CO_2_ and C_2_H_4_), ↓ microbial growth, ≃ TSS, ↑ polysaccharides, ↑ antioxidant properties (just the coating with phenolics), ↓ Vit C reduction, and ≃ sensorial parameters	[143]
CMC–bacterial cellulose	Olive and ginger oil	Casting	Oranges and tomatoes (dipping)	Film: ≃ WS, ≃ moisture, ↓ TS, ↑ EB, non-toxic toward NIH-3T3 fibroblasts, and antimicrobial activity Fruits: ↓ WL, ↑ overall acceptability along storage time, and ↑ SL (films containing ginger oil were more effective)	[218]
Ethyl cellulose	Medium chain triglyceride oleogel	Hot extrusion 3D food printer	NA	↑ oil binding capacity (↑ ethyl cellulose). PEG addition: ↑ overall printability, ↑ plasticity, ↑ viscosity, ↑ storage modulus, ↑ gel strength, ↑ shear thinning behaviour, and ↓ G’ in melting point (45 °C)	[219]
Xanthan gum–CMC	ZnO-NPs	Casting	Tomatoes (dipping)	Films: ↑ opacity, ↑ thermal stability, ↓ WVP, ↑ TS, ↓ EB, and ↑ antimicrobial activity Tomatoes: ↓ WL and ↑ SL	[220]
Chia seed mucilage–bacterial CNF	NA	Coating dispersion	Strawberries (dipping)	↑ TPC, ↑ TFC, ≃ TAC, ↑ Vit C, ↑ antioxidant activity, ↓ PO activity, and ↓ peroxidase enzymes activity	[221]
Alginate	Hydroxyapatite–quercetin	NA Solubilization	Chicken fillets (dipping, layer by layer)	↓ release rate (compared to free quercetin), ↑ L* preservation, ≃ ΔE, ↓ WHC, ↑ antibacterial activity, ↓ harness increase, ↓ springiness reduction, ↓ gumminess and chewiness decrease, ↓ TVB-N, and ↑ overall acceptability throughout storage time	[222]
Alginate, chitosan, and soy protein isolate	NA	Coating dispersion	Mozzarella cheese (dipping, enrobing, spraying, and electrostatic spraying)	↓ WL (> chitosan > alginate >soy protein), ≃ hardness, ↓ WL (dipping > enrobing > spray > electrospray), ↓ thickness (spray and electrostatic spray), ↑ homogeneity (dipping and enrobing), and ↑ spreadability (alginate)	[144]
Alginate	NA	Coating dispersion	Melon (dipping and vacuum impregnation)	↑ firmness (vacuum impregnation), ↑ weight gain (vacuum impregnation), and ↑ ΔE (vacuum impregnation)	[152]
Alginate	*Aloe vera* and Frankincense oil	Casting	Green capsicums (wrapped)	Films: ↑ thermal stability, ↑ TS, ↑ EB, ↑ brightness, ↑ yellowness and greenness, ≃ transparency, ↑ UV-shielding, ↓ WVP, and ↓ microorganism growth Green capsicums: ↓ senescence and ↓ WL	[223]
Alginate	*Brassica juncea* extract and *Raphanus sativus* sprout extract	Casting (zein-chitosan microparticles containing the extracts)	Tomatoes (dipping)	Films: ↑ thermal stability, ↑ hydrophobicity, ↓ water absorption, ↓ WCA, ↓ TS, ↑ EB, ↓ WVP, ↑ antibacterial activity, and ↑ antioxidant capacity Tomatoes: ≃ appearance, ≃ texture, and ↑ SL (30 days)	[224]
Alginate	Myrtle, rosemary extract, irradiation	Coating dispersion	Beef cattle meat (dipping and packed PP trays)	↓ lipid oxidation, ↑ antioxidant properties, ↓ L*, ↑ redness, ↓ firmness, ≃ pH, ↓ microorganism growth, and ↑ SL (6 days) (most effective treatment: irradiation and coatings with myrtle extract)	[225]
Alginate	*Citrus sinensis* EO	Nanoemulsion	Tomatoes (dipping)	↑ whiteness index, ↑ antibacterial activity, ↑ firmness, ↓ WL, ↓ pH increase, and ↑ overall acceptability	[75]
Alginate–chitosan	Fucoidan	Coating dispersion	Rainbow trout fillets (dipping. Layer-by-layer)	↑ fish quality, ↓ lipid oxidation, ↓ TVB-N, ↑ antibacterial activity, ↑ overall acceptability along storage time, and ↑ SL (10 days)	[226]
Glucomannan–*κ*-carrageenan	*Salmonella enteritidis* phage PBSE191	Casting (Hydrogel film)	Chicken meat (wrapped)	Film: ↑ TS, ↓ EB, ↑ water swelling ratio, ↑ moisture content, ↓ WS, and ↓ WVP Chicken meat: Salmonella-killing (↓ bacterial growth)	[227]
ι and κ-carrageenan—high and low methoxyl pectin	Tomato paste	Casting	NA	red colour films, ↑ WVP, ↓ moisture ↑ weight, ↑ opacity, ↑ strength, ↑ flexibility, ↑ stiffness, and ↑ antioxidant properties	[228]
κ-carrageenan	Dill EO–oxygen absorber	Coating dispersion	Chilled rainbow trout fillets (dipping)	↓ pH increase, ↓ TVB-N, ↓ conjugated dienes, ↓ lipid oxidation, ↓ protein carbonyls, ↓ electric conductivity, ↓ organoleptic deterioration, ↑ antioxidant properties, ↑ antibacterial properties, and ↑ SL (16 days)	[229]
Konjac glucomannan–carrageenan	Camellia oil	Coating dispersion	Chicken meat (dipping)	↓ WL, ↓ pH increase, ↓ lipid oxidation, ↓ TVB-N, ↑ antibacterial properties, ↑ overall acceptability along storage time, and ↑ SL (10 days)	[230]
Tragacanth gum–carrageenan	Clove EO	Casting	NA	↓ TS, ↓ YM, ↑ EB, ↓ moisture content, ↑ WS, ↓ WVP, ↑ ΔE, ↑ antibacterial properties, and ↑ antioxidant capacity	[231]
κ-carrageenan	Spent coffee grounds oil	Casting (emulsion)	NA	↓ TS, ↑ EB, ↑ WS, and ↑ antioxidant capacity	[232]
Pullulan	*Auricularia auricular* extracts	Casting	Potato fresh cut (dipping)	Films: ↓ transparency, ↑ compactness, ↑ thermal stability, ↑ WVP, ↑ WS, ↑ TS, ↑ EB, ↑ ΔE, ↑ antioxidant properties, and ↑ antimicrobial properties Potato: ↓ browning index, ↓ microbial growth, ↑ TSS, ↓ WL, and ↑ SL	[89]
Pullulan–gelatin	Clove EO	Casting (nanoemulsion and pickering emulsion using WPI and inulin)	NA (release assay in 95% EtOH)	↑ antibacterial properties, ↓ density (NE) and ↑ with emulsion, ↓ moisture content, ↓ WVP, ↓ TS, ↓ EB (NE) and ≃ with emulsion, ↓ ΔE (NE) and ↑ with emulsion, ↑ antioxidant properties, emulsion and ↓ release rate after 72 h	[233]
Pullulan–chitosan	Galangal EO	Casting	Mango (dipping)	Films: ↓ WVP, ↑ WS, ↑ TS, ↑ thermal stability Mangoes: ↓ WL, ↓ firmness decrease, ↑ titratable acidity, and ↓ TSS	[234]
Agar–agar	Bacteriocin of *Lactobacillus sakei*	Casting	Curd cheese (covered)	Films: ↓ WVP, ↑ WS, ↑ TS, ↑ EB, ↓ thermal stability, and ↑ antibacterial activity Cheese: ↓ bacterial growth	[235]
Agar–gelatin	*Aloe vera* EO	Casting	Kashar cheese (wrapped)	Films: ≃ WC, ↓ WS, ↑ antioxidant properties, and ↑ antimicrobial properties Cheese: ≃ pH and ↓ colour variance	[236]
Levan–pullulan–chitosan	ε-polylysine	Casting	Strawberries (dipping)	Films: ↓ WVP, ↓ oxygen permeability, ↑ WCA, ↓ TS and ↑ EB (↑ levan-pullulan ratio), ↑ opacity, ↑ ΔE, ↓ L*, ↑ moisture content, ↓ WS, and ↑ antimicrobial properties Strawberries: ↓ WL, ↓ firmness decrease, and ↓ TSS	[86]
Pectin–pullulan	Grape seed extract	Casting	Peanuts (dipping)	Films: ↓ L*, ↑ ΔE, ↑ UV-blocking properties, ↑ TS, ↓ EB, ≃ WVP, ≃ WCA, ↑ antibacterial properties, ↑ antioxidant properties Peanuts: ↓ peroxide value, ↓ lipid oxidation, ↑ SL	[237]
Xanthan gum	Betel leaf extract	Coating nanoemulsion	Tomatoes (dipping)	NE: ↑ viscosity (with ↑ xanthan gum), ↑ antimicrobial activity Tomatoes: ↓ WL, ↓ pH increase, ↓ TSS, ↓ firmness decrease, ↑ Vit C, ↑ TPC, ↑ antioxidant capacity, ↑ overall acceptance along storage time, ↓ microorganism growth, and ↑ SL (6 days)	[101]
Persian gum–gelatin	Thyme EO	Casting (Pickering emulsion)	Barred mackerel fillet (dipping)	Films: ↓ moisture content, ↓ WVP, ↓ TS, ↓ EB (4% of emulsion), and ↓ opacity (4% of emulsion) Mackerel: ↓ pH increase, ↓ lipid oxidation, ↓ microorganism growth, and ↑ sensory attributes perception	[238]
Guar gum	*Butea menosperma* flower extract	Casting	Tomatoes (dipping)	Films: ↓ TS, ↑ EB, ↓ L*, ↑ ΔE, ↑ thermal stability, and ↑ antioxidant properties Tomatoes: ↓ firmness reduction, ↓ WL, ↓ TSS, ↓ decay, ↑ acceptability, and ↑ SL	[239]
Alginate–algaroba seed galactomannans/cashew gum–gelatin	NA	Coating dispersion	Grapes ‘Italia’ (dipping)	↓ WL, ↓ firmness reduction, ↓ TSS, ≃ L*, ≃ pH, ↑ TPC, and ↑ antioxidant properties (2% alginate, 0.5% glucomannans, 0.5% cashew gum)	[240]
Kefiran–gelatin	*Zhumeria majdae* EO	Casting (nanoemulsion)	Sponge cakes (packaged)	Films: ↓ WVP, ↓ TS, ↑ EB, ↓ L*, ↑ ΔE, ↑ YI, ↓ light transmission, ↑ opacity, ↑ thermal stability, ↑ antioxidant properties, and ↑ antimicrobial capacity	[83]
Chia seed mucilage–bacterial CNF	NA	Coating dispersion	Strawberries (dipping)	≃ TAC, ↑ vit C, ↑ TPC preservation, ↑ antioxidant properties, ↓ PO, ↓ PA, ↓ SOD, and ↓ ALP	[221]
*Plantago major* seed mucilage	*Citrus limon* EO	Coating dispersion	Buffalo meat (dipping)	↓ PO (↓ lipid oxidation), ↓ microbial growth, ≃ pH, ↓ moisture reduction, ↓ hardness reduction, ↓ ΔE, and ↑ overall acceptance	[241]
Fenugreek seed mucilage–CMC	Rosemary EO	Casting	Apples (dipping)	Films: ↓ moisture content, ↑ WVP, ↓ WS, ↓ L*, ↑ ΔE, ↓ light transmission, ↑ transparency, ↓ thermal stability, ↓ TS, ↓ EB, ↑ TPC, and ↑ antibacterial activity Apples: ↓ ΔE and ↑ SL	[242]
MHEC–brewer’s spent grain arabinoxylans	NA	Casting	NA	↓ TS, ↑ thermal stability, ↑ hydrophobicity (↑ WCA), ↓ moisture content, ↓ WS, ↑ WVTR, and ↑ antioxidant properties	[243]
Brewer spent grain arabinoxylans–CNF	Ferulic acid or feruloylated arabinoxylo-oligosaccharides	Casting	NA	↑ UV-blocking properties, ↓ YM, ↓ TS, ≃ EB, ↑ thermal stability, ↑ antioxidant properties, and ↑ antimicrobial activity	[244]
Arabinoxylan	Tea polyphenol	Casting	Grapes (dipping)	Films: ↓ light-transmittance, ↑ TS, ↓ EB, ↓ WVTR, and ↑ antioxidant properties Grapes: ↓ transpiration of water, ↓ WL, ↑ titratable acids, ↑ vit C, ↓ shrivelling rates, and ↓ spoilage	[245]
Water extractable arabinoxylans	NA	Casting	Cherry and strawberry (dipping)	Films: ↑ flexibility, ↑ WVP, ↑ thermal stability, ≃ TS, ≃ EB, and ≃ WVP Fruits: ↓ WL, ↓ colour degradation, ↓ decay, ↓ TSS, ↓ TAC loss, ↓ softening process, ↓ vit C decline, ↓ malondialdehyde content, and ↑ SL (more effective on cherries)	[246]
Carboxymethylated tamarind seed polysaccharide	ε-Polylysine	Casting	Green Bell Pepper (dipping)	Films: ↑ WVP, ↓ WCA, ↓ TS, ↓ EB, ↑ antioxidant properties, and ↑ antimicrobial activity Pepper: ↓ WL, ↓ malondialdehyde content, ↓ hardness decrease, ↓ vit C loss, ↓ nutrients reduction, and ↑ SL	[247]
Tamarind xyloglucan/protein–chitosan	NA	Casting	NA	↑ thermal stability, ↓ TS, ↑ EB, ↑ MC, ↓ WS, and ↓ swelling degree	[248]
Glutenin–tamarind gum	Melatonin-pummelo EO	Casting (microemulsion)	White mushroom (covered)	Films: ↓ thermal stability, ↑ TS, ↑ EB, ↑ OP (just with EO), ↓ WVP, ↑ UV-blocking properties, and ↑ antioxidant properties Mushroom: ↑ overall acceptability, ↓ respiration rate, ↓ malondialdehyde content, and ↑ SL	[249]
Tamarind xyloglucan–starch	Lignin nanoparticles	Casting	Banana (dipping)	Films: ↑ TS, ↑ EB, ↓ YM, ↑ WCA, ↓ WVP, ↑ TPC, and ↑ antioxidant properties Banana: ↑ UV-blocking properties, ↓ WL, and ↓ colour change	[250]
Shellac	Tannic acid	Coating dispersion	Mangoes (dipping)	↓ WL, ↓ firmness reduction, ↓ respiration rate, ↓ browning index, ↓ lipid oxidation, ↓ TSS, ↑ TPC, ↑ antioxidant properties, ↓ membrane permeability, ↓ enzymatic activity, ↓ vit C loss ↑ volatiles preservation, ↑ antifungal activity, ↑ overall quality, and ↑ SL (10 days)	[251]
Shellac–soy protein-starch	Juglone from walnut green husk extract	Coating dispersion	Wichita pecans (smeared)	↓ pure kernel loss, ↓ acid value increase, ↓ fat loss, ↑ PO, ↑ overall taste, ↑ interior brown colour, and ↑ antibacterial activity (combination of shellac, soy protein, and starch with juglone)	[252]
Zein–shellac	Curcumin	Casting	NA	↓ WVP, ≃ WS, ↓ EB, ↑ TS (1, 3% curcumin), ↓ TS (5, 7% curcumin), ↑ WCA, ↓ L*, ↑ ΔE, ↑ YI, ↑ opacity, ↓ thermal stability. Controlled release, pH responsiveness, ↑ antioxidant properties, and ↑ antibacterial properties	[253]
Shellac	NA	Electrospinning	(coating on paper to package tomatoes)	Coating: ↑ TS, ↓ WVTR, ↓ OP, ↓ oil permeability, ↑ moisture resistance, ↑ paper integrity (↓ roughness, ↑ homogeneity) Tomatoes: ↓ WL, ↓ citric acid decrease, and ↓ pH increase	[254]
Pectin	*Ilex paraguariensis* extract	Casting	NA	≃ WVP, ↓ L*, ↑ ΔE, ↑ TPC, ↑ antioxidant properties, ↑ thermal resistance, and ↑ UV blocking properties (10% extract, 15% sorbitol optimum, compared to control film)	[255]
Pectin–beeswax	*Satureja montana*, *Cinnamomum zeylanicum*, *Commiphora myrrha* EOs, eugenol, geraniol, vanillin, and propolis extract	Coating dispersion	“Valencia” oranges (rubbing)	↓ fungal growth, ↓ WL (*Commiphora myrrha* most effective), ↑ SL, ≃ firmness, and ≃ titratable acidity Antifungal properties: Vanillin > propolis extract > *Satureja montana* > *Cinnamomum zeylanicum* > eugenol > geraniol > *Commiphora myrrha*	[256]
Low-methoxyl pectin	Epigallocatechin gallate	Lyophilization (free radical grafting method)	Grapes (dipping)	Coatings: ↓ thermal stability, ↑ antioxidant properties, and ↑ antibacterial properties Grapes: ↓ WL, ↑ TPC, ↓ firmness reduction, ↓ polyphenol oxidase activity, ↓ malondialdehyde content, ↓ lipid oxidation ↓ microorganism growth, ↑ wetting, ↑ water adhesion, and ↓ water spreading	[257]
Pectin	Carvacrol/2-hydroxypropyl-β-cyclodextrin inclusion complex	Casting	NA	cyclodextrin ↑ carvacrol solubility and stability, ↓ viscosity, ↑ WCA, ↑ thermal stability, and ↑ antifungal activity	[258]
*Others*					
NA	Jaggery	NA	Apple snacks (vacuum impregnation and osmotic dehydration; freeze-drying and hot air-drying)	↓ L*, ↑ ΔE ↑ antioxidant capacity, and ≃ TPC Freeze-drying: preserves the antioxidants Convective hot air drying: ↑antioxidant properties	[151]
NA	Enzymes (polygalacturonase, pectin methylesterase and pectin lyase)	Coating dispersion	Pumpkin (vacuum impregnation)	↓ stiffness values, no fracture point, homogeneous texture profile, ↑ antioxidant capacity, and ↓ total and reducing sugars	[150]
NA	Turmeric extract	NA	Jasmine white rice kernels (Top-Spray Fluidized Bed Coating)	Recycling of 80% exhaust air ↓ fissured kernels (<11.8% moisture content) and saved the energy consumption (41.7–46.5%). Coating efficiency 75–86%. Head-wrapped yield 94–95%	[259]
NA	Rosemary extract	Encapsulation with maltodextrin-gum arabic-WPC	Microcrystalline cellulose cores (fluidized bed coating)	Coating efficiency: 64.3–79.2. Agglomerates 0.2–47.7. Layering > agglomeration as the main growth mechanism of MCC cores. Retention efficiencies ≃ 70% (except for caffeic acid, ≃ 60%)	[159]
^a^ Chitosan–graphene oxide nanofiller	NA	NA Coating dispersion	PBS (ultrasonic spray coating, layer-by-layer)	↑ moisture resistance, ↑ mechanical and scratch resistance, ↓ OP, ↓ CO_2_ permeability, ↓ swelling, and ↓ light transmission	[260]
Defatted soybean meal	NA	Casting (cold plasma treatment with O_2_-, N_2_-, air-, He-, and Ar-)	Smoked salmon (packed)	Ar-treatment: ↑ EB, ↑ TS, ↓ WVP, ↑ L*, and ↑ ink adhesion (15 min, 400 W) Salmon: ↓ lipid oxidation, ↓ Hue angle reduction, and ↓ hardness reduction	[261]

^a^ Not edible, but the only scientific paper found that has used ultrasonic spray coating in food packaging. WPC: whey protein concentrate; WPI: whey protein isolate; CMC: carboxymethyl cellulose; CNC: cellulose nanocrystals; CNF: cellulose nanofibers; PVA: polyvinyl alcohol; MHEC: methyl hydroxyethyl cellulose; NP: NP; EO: essential oil. VI: vacuum impregnation; TS: tensile strength; EB: elongation at break; YM: YM; L*: lightness; YI: yellowness index; SL: shelf life; TVB-N: total volatile basic nitrogen; WL: weight loss; TPC: total phenolic content; TFC: total flavonoid content; TAC: total anthocyanin content; WS: water solubility; WCA: water contact angle; WHC: water holding capacity; WVP: water vapour permeability; WVTR: water vapour transmission rate; ΔE: total colour difference; TSS: total soluble solids; PO: polyphenol oxidase; PA: peroxidase activity; SOD: superoxide dismutase activity; ALP: Ammonia-lyase phenylalanine activity.

## 4. Controlled Release in Active Edible Coatings and Films

Recently, active packaging has attracted great attention as a carrier for controlling the release of bioactive species, such as antimicrobial or antioxidant agents [262]. The controlled release of active agents through food packaging materials is a novel release technology in which the active compound is released in a controlled manner over time, ensuring the desired concentration on the surface of the food, thereby performing its function, maintaining the quality of the food product, and extending its shelf life [263,264]. Among controlled-release packaging types, edible coatings have gained widespread attention [264]. Coating represents a key method for producing controlled-release active packaging, where food products are coated with a thin layer of biopolymers with bioactive-loaded particles [265]. A variety of functionally bioactive compounds can be incorporated into the active films and coatings to be used as active packaging materials and vehicles to deliver key compounds [266], including phenolic compounds, probiotics, and prebiotics [6].

**Figure 2:**
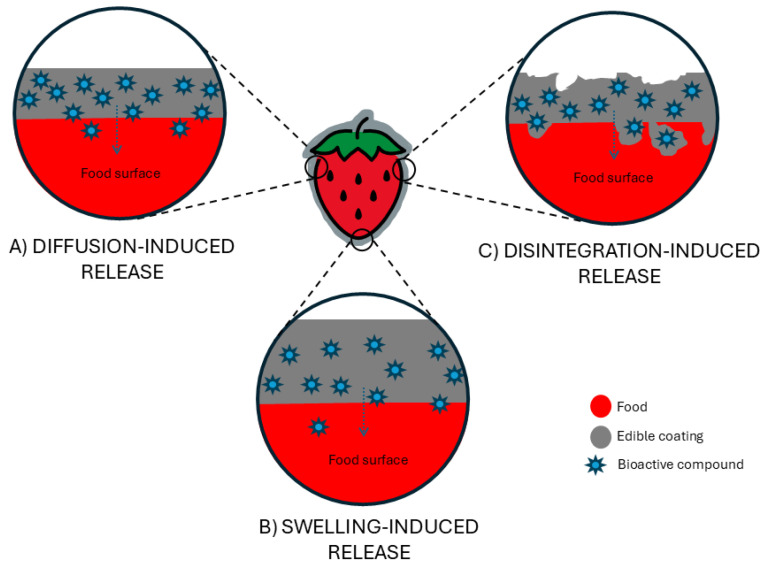
Controlled-release mechanisms of bioactive compounds from edible coatings: (**A**) illustration of diffusion; (**B**) swelling; and (**C**) disintegration processes.

One of the principal factors in the development of controlled-release active packaging is determining the release mechanism [267]. Three types of release mechanisms have been proposed for active packaging systems incorporated directly into food (Figure 2):

1. Diffusion-induced release: volatile bioactive compounds diffuse from the packaging into the food through air or non-direct contact spaces. This mechanism is common in petroleum-based and water-resistant polymers.
2. Swelling-induced release: bioactive compounds are released when moisture-sensitive packaging material, such as polypeptides or polysaccharide-based films, swells, allowing the bioactive compounds to be released by direct contact.
3. Disintegration-induced release: applicable to biodegradable or reactive non-biodegradable polymers where the packaging material partially breaks down releasing the active compounds [263,264,267].

The diffusion process is the main phenomenon in the release of bioactive compounds from active packaging, generally described by Fick’s second law [267]. The main challenge is to control the rate or kinetics of the release of the active compounds during the shelf life of the food: too slow a release may not provide sufficient bioactive agents at the beginning of storage, while too rapid a release may deplete active agents before the end of shelf life [263,264,265,267].

Mathematical modelling based on Fick’s law, together with the diffusion coefficient (D) and the partition coefficient (K), provides a useful framework to predict and optimize controlled release. These parameters describe how molecules migrate through the packaging material and how they distribute between the polymer matrix and the food or simulant [231]. Food simulants, established by Commission Regulation (EU) No. 10/2011 [268], are often employed to facilitate such studies [269].

However, mathematical models present limitations in real food applications since the release of active compounds is affected by certain conditions, including internal factors (bioactive agents, polymer matrix, and active layer) and external factors (storage and product characteristics) [262,264,267,270,271]. For example, larger bioactive agents have a slower release rate, while polymers with higher porosity or storage at elevated temperatures accelerate release [89,103]. Additionally, the pH of the medium can significantly alter release behaviour, as observed for rosemary essential oil and carvacrol [264,272].

Table 3 shows some examples of controlled release in active edible coatings and films. Pinheiro et al. (2012) [273] investigated and revealed how the spatial position of methylene blue within a κ-carrageenan/chitosan nanolayered coating, as well as the environmental conditions (pH and temperature), significantly affected the release profile, highlighting the importance of these factors in designing controlled-release systems. The study by Mastromatteo et al. (2009) [274] also showed that thymol release from a zein-based film decreased with increasing film thickness, while higher spelt bran content significantly accelerated its release. Arcan et al. (2014) [275] found that the use of fatty acids with different chain lengths in the production of zein blend films affected the release rate of lysozyme and catechin, while changing the number of double bonds in the fatty acids affected only the release rate of catechin.

**Table 3:** Application of bioactive compounds in controlled release systems using edible polymer matrices, with encapsulation methods, food product or food simulant applications, and observed food effects or controlled-release behaviour.

Bioactive Compound	Coating Matrix/ Polymer	Encapsulation/ Controlled-Release Method	Food Product/Food Simulant	Observed Effect/Benefit	Reference
Thymol	Zein	Mono and multilayer films	Distilled water	Thymol release can be controlled by varying the thickness of the layers and the amount of biodegradable fibre	[274]
Aroma compounds (methyl-ketones, ethyl-esters, alcohols)	ι-carrageenan	Emulsified film with lipid globules	Not applicable	Improved retention of polar aroma compounds and gradual release over time	[276]
Methylene blue	Κ-carrageenan and chitosan on polyethylene terephthalate	Multilayer nanocoating via layer-by-layer deposition	PBS of a certain pH (2.0 or 7.0) and temperature (4 or 37 °C)	Controlled and modulable release behaviour, adjustable according to pH, temperature, and incorporation layer	[273]
Riboflavin and α-tocopherol	Whey protein with gum arabic, low-methoxyl pectin or κ-carrageenan	Water-in-oil-in-water (W/O/W) microcapsules	HCl solution (pH 1.2); simulated gastric condition (pH 1.2) with 0.1% pepsin; phosphate-buffered saline (pH 7.4); and simulated intestinal condition (pH 7.4) with 1.0% pancreatin	Gum arabic showed better control of the liposoluble vitamin (α-tocopherol), while κ-carrageenan showed better control of the water-soluble vitamin (riboflavin).	[277]
Lysozyme, catechin, and gallic acid	Zein–wax composite	Aggregated hydrophobic wax particles	Fresh Kashar cheese	All lysozyme-containing films inhibited *L. monocytogenes* growth for 8 weeks, while films with catechin and gallic acid effectively prevented oxidative changes	[278]
Lysozyme and (+)-catechin	Zein blended with oleic, linoleic, or lauric acids (with lecithin)	Microsphere encapsulation	Water	The use of fatty acids with varying chain lengths affected the release rates of both compounds, whereas changes in the number of double bonds influenced only the release of catechin	[275]
Rosemary oil	Mucilage	Liposomes	Fresh-cut banana	The liposomal oil combined with mucilage inhibited polyphenoloxidase, lipoxygenase activities, fruit softening and weight-loss, and retained higher firmness and soluble solids content	[279]
β-carotene	Xanthan gum	Nanocapsules and nanospheres; Korsmeyer–Peppas and Higuchi matrix-type models	Fresh-cut melon (*Cucumis melo*, var. cantaloupe)	Improved β-carotene retention was achieved with minimal changes in whiteness and firmness helping to extend shelf life to 21 days at 4 °C	[280]
Folic acid	Alginate/chitosan	Nanolaminated films by the layer-by-layer technique and post-diffusion	Phosphate-citrate buffer solutions at pH 3 or 7	The release profiles were affected by pH conditions, showing a greater release in small intestine pH conditions where it is supposed to be adsorbed	[281]
Curcumin	Carboxymethylated filter paper	Nano metal–organic frameworks	Pitayas	On day 6, the rotten areas of the cut treated were below 5%, exhibiting a superlong-acting performance	[282]
Spent black tea (SBT) extract	Pectin–sodium caseinate in a cassava starch matrix	Microencapsulation	Water and 95% ethanol	Microencapsulation of SBT protected its antioxidants during film processing and significantly enhanced their migration into both simulants	[283]
Curcumin	Chitosan	Hydrogen bonding interactions	Litchis, strawberries, mangos, and plums	This coating enabled long-term release (up to 38 days) of natural preservatives on the surface of the fruit, maintaining freshness and appearance at least 9 days longer than uncoated samples	[284]
*Angelica archangelica* essential oil	Chitosan	Nanoemulsion	Grapes (*Vitis vinifera* L.)	Inhibited the contamination of *Botrytis cinerea*; preserved weight, acidity, total soluble solids, phenolics, pH, enzymatic antioxidants; reduced respiration rate; and enhanced sensory quality over 30 d of storage	[285]
Cinnamon essential oil (CEO)	Chitosan/gelatin	Pickering emulsion	50% ethanol and 95% ethanol	Improved antimicrobial activity, enhanced thermal and mechanical stability, and exhibited better barrier properties and controlled release of CEO	[286]
Thyme essential oil	Whey protein	Co-precipitation in β-cyclodextrin	Water and ethanol 95%	Encapsulation improved volatile retention in the film and enabled gradual release, with slower rates in water compared to 95% ethanol	[287]
Thyme essential oil	Chitosan	Pickering emulsion	Strawberries	The emulsion effectively prevented weight loss, reduced firmness decline, inhibited pH increase, decreased titratable acidity, and restricted microbial growth	[288]
Vanillin	Chitosan	2-hydroxypropyl-β-cyclodextrin; pH-dependent controlled release	Chicken	It extended shelf life and maintained sensory quality by delaying pH increases, preventing the proliferation of microorganisms, and inhibiting lipid oxidation	[289]
Thyme essential oil	Chitosan and carboxymethyl cellulose	Water-in-oil nanoemulsions	Strawberries	Potent antimicrobial effect, effectively controlling the growth of *Botrytis cinerea* and maintaining fruit quality (significantly reduced weight loss, preserved firmness) for 15 days at 4 °C	[290]
Whey isolate protein fibre and glycyrrhizic acid (3:1)	Sodium alginate	Temperature-responsive emulsion system	Grapes	Maintained pH, soluble solids, and vitamin C; reduced weight loss by 44.5% at 45 °C after 6 days	[291]
Thymol	Alginate	Nanoemulsion and nanostructured lipid carriers	Carrot	Higher peroxidase activity, total phenolic content, flavonoid content, DPPH radical scavenging activity, pH, and lower respiration rate, total soluble solids, weight loss, and decay, particularly with nanostructured lipid carriers	[292]

When conducting experimental tests, it is very important to design them correctly and to consider that the release depends entirely on the compatibility of the bioactive compound and the selected food product or food simulant. However, it was found that most studies focus on the development and physicochemical characterization of active edible coatings and films by scanning electron microscopy, Fourier transform infrared spectroscopy analysis, zeta potential, particle size, etc., but only a few of them evaluate the release of the active compounds through testing with food simulants or in the foods themselves. For example, Rajapaksha and Shimizu (2021) showed a significantly higher release of antioxidant compounds from spent black tea microcapsule films in water than in 95% ethanol [283]. Fan et al. (2023) [286] evaluated the controlled release of cinnamon essential oil from gelatin/chitosan films stabilized with Pickering emulsion in 50 and 95% ethanol solutions, simulating semi-fatty and fatty foods, respectively. The release rates depended on the concentration of the essential oil in the matrix, showing that adjusting its content makes it possible to modulate the release. In the system with 50% ethanol, the higher amount of water caused swelling and the partial dissolution of the film, weakening the network structure and increasing the release [286]. Edible whey films incorporating thyme essential oil encapsulated in β-cyclodextrin inclusion complexes demonstrated a gradual release of volatiles, with significantly higher release rates in 95% ethanol than in water, indicating controlled and matrix-dependent release behaviour relevant to food packaging applications [287]. Some studies have investigated controlled release in the gastrointestinal fluids themselves. Acevedo-Fani et al. (2018) [281] developed edible alginate/chitosan nanolaminates loaded with folic acid, designed for pH-dependent controlled release. Release was tested in simulated gastrointestinal conditions (phosphate–citrate buffers at pH 3 and 7, 37 °C for 7 h) and the nanolaminates exhibited minimal release at acidic pH values and a pronounced release at a neutral pH. These results suggest that the system enables targeted folic acid delivery, protecting it in the stomach and promoting its release in the small intestine, the primary site of absorption. Liu et al. (2013) [277] prepared a film-forming emulsion of polysaccharide and whey protein containing both liposoluble and water-soluble vitamins. Different polysaccharides such as gum arabic, low-methoxyl pectin, and κ-carrageenan showed distinct synergistic effects with whey protein, resulting in different results regarding the controlled-release properties of the vitamins in simulated gastrointestinal conditions. Gum arabic showed better control of the liposoluble vitamin (α-tocopherol), while κ-carrageenan showed better control of the water-soluble vitamin (riboflavin).

Table 3 also shows several studies that conducted shelf life studies with real foods coated with active edible coatings or films. As can be seen in the table, most are applied to fresh fruits (such as banana, litchis, strawberry, mango, plum, pitaya, grape, and melon), but there are also studies on other foodstuffs like cheese, chicken, and vegetables. Various natural polysaccharides have been used as polymeric matrices, since they are renewable resources with excellent biocompatible, degradable, and antimicrobial properties [246], with chitosan being the most common. Conventional systems such as liposomes, nanoemulsions, and modified cyclodextrins have been used, as well as more advanced technologies such as Pickering emulsions, metal–organic frameworks, and lipid nanostructures. These techniques allow the release to be tailored to environmental factors such as pH, temperature, or the polarity of the medium. Kinetic models such as Korsmeyer–Peppas and Higuchi have been useful to describe the dynamics of β-carotene release through xanthan gum coatings combined with nanocapsules [280].

In some cases, edible coatings are intended to impart flavour by encapsulating and gradually releasing aromatic compounds, particularly the more polar volatiles that are typically evaluated by gas chromatography–mass spectrometry (GC-MS) [277]. For example, Marcuzzo et al. (2010) [276] developed edible films based on a ι-carrageenan emulsion that managed to retain volatile compounds during the film formation process and gradually release them over time.

Studies on the release of bioactive compounds from active and edible coatings are still scarce, so it is important to continue researching how the film matrix, the carrier particle, and the type of bioactivity affect this release, as well as to investigate the mechanisms involved and develop mathematical models that enable the description and prediction of the release profile [264].

## 5. Functional Food Applications

Edible coatings and films are a sustainable and effective method of preserving food and increasing its shelf life by acting as passive barriers that alleviate the need for chemicals, protecting products from moisture, oxidation, mechanical stress, and microbial infestation [293]. As shown in Table 4, numerous studies demonstrate the potential of edible coatings and films not only to extend the shelf life of foods—such as vegetables, fruits, fish, meat, cereals and their derivatives, and dairy products—but also to enrich their content in bioactive compounds, thus improving their functionality and health benefits (functional foods) including antioxidant, anti-inflammatory, and cardioprotective effects; improving gut and immune health; aiding in disease prevention; and enhancing nutrient absorption, among other effects [6,294]. Edible coatings/films may include bioactive compounds transforming food products into “functional” foods, which can be defined as “foods that beneficially affect one or more specific functions of the body, beyond the proper nutritional effects, in a manner that is relevant to improved health and well-being and/or a reduction in disease risk” [294]. Among these compounds, polyphenols stand out due to their antioxidant, anti-inflammatory, and antimicrobial functions, which not only help protect food quality but may also confer health benefits after long-term storage. These natural plant-derived molecules can modulate oxidative stress and are associated with a reduced risk of chronic diseases such as cancer, diabetes, osteoporosis, neurodegenerative diseases, and cardiovascular disease. In addition to polyphenols, other compounds such as vitamins, minerals, probiotics, and prebiotics, among others, may also be incorporated depending on the functional goal of the coating (Table 4) [295,296]. However, due to their chemical and physical properties, many bioactive substances cannot be introduced into the food system in a simple free state [295]. Their inclusion in edible films and coatings is a way to increase their viability and survival during food production processes and to reach the gastrointestinal tract in sufficiently large quantities so that they can be effective [294]. For example, El-Sayed et al. (2021) [297] developed an ecological probiotic edible coating based on chitosan, sodium alginate, and carboxymethylcellulose with probiotic strains (*Bifidobacterium lactis*, *Lactobacillus acidophilus*, and *Lactobacillus casei*) to improve the shelf life of ultrafine soft cheese for 45 days, thus achieving a functional product with antimicrobial properties. Semwal et al. (2022) [298] developed a sodium caseinate-based edible probiotic film for wheat buns with chia mucilage as a protectant to improve the viability of probiotic bacteria (*Limosilactobacillus fermentum* NKN51 and *Lactobacillus brevis* NKN52) for 3 weeks at 4 °C and 2 weeks at 25 °C.

However, sometimes, after intestinal simulation, the probiotic load decreases sharply due to the change in pH and the presence of different enzymes that can affect the integrity of the coating [294]. In these cases, prebiotics can also increase the viability of probiotics in films [299]. For example, Sáez-Orviz et al. (2020) [294] designed symbiotic bioactive coatings combining lactobionic acid as a prebiotic and *Lactobacillus plantarum* as a probiotic to produce a novel functional dairy product. The results showed that probiotic levels remained within acceptable ranges after simulated digestion, thanks to the protective effect of lactobionic acid. In another study, Alvarez et al. (2021) developed a new non-dairy probiotic food by adding an alginate-based solution enriched with prebiotics (inulin and oligofructose) and probiotic cultures (*Lactobacillus rhamnosus* and *Bifidobacterium animalis subsp. lactis*), which resist simulated gastrointestinal digestion conditions, to fresh-cut apples [300].

In addition, it is essential to conduct studies that confirm the ability of active compounds to diffuse through coatings and reach the food matrix, as demonstrated by Tampucci et al. (2021) [301] in their research on tyrosol-enriched tomatoes. The authors evaluated a coating based on chitosan, and found that tyrosol, a hydrophilic molecule associated with health benefits, could pass through the tomato peel and infiltrate the pulp, maintaining constant levels for seven days of storage [301].

Edible coatings are also used in combination with osmotic dehydration to develop healthy and sustainable fruit and vegetable snacks with improved properties, as they allow the removal of water without significant nutrient losses, optimizing the texture and physicochemical properties [302].

Although studies on functional food applications are limited, there is considerable interest in the potential of nanocomposite systems as carriers of bioactive compounds to improve edible coatings, enhance the controlled release and dispersibility of bioactives within the food matrix, and increase the overall functionality of food systems [296]. Khatreja & Santhiya (2024) [303] developed an oral disintegrating film (ODF), a convenient and friendly alternative for patients with swallowing difficulties, composed of hyaluronic acid, okra mucilage, suspended vitamin C-loaded bioactive glass nanoparticles, and clove essential oil as a potential functional food. The developed ODF showed antibacterial, antioxidant, and hemocompatible properties. The incorporation of nanoparticles and essential oil improved the thermal and mechanical properties and porous nature of the films, making them useful for treating mouth ulcers, which also alleviates the pain due to the effect of the eugenol in clove oil [303].

**Table 4:** Recent advances in the use of functional compounds for the development of edible coatings and films materials with food applications, functional properties, and health benefits.

Functional Compound	Coating/Film Material	Functional Property	Food Product	Health Benefit	Reference
Probiotic (*Lactobacillus plantarum* CIDCA 83114) and prebiotic (fructooligosaccharides)	Methylcellulose	Symbiotic delivery increases the viability of probiotics after both 90 days of storage and contact with a simulated gastrointestinal environment, and maintain sensory properties	Apple snacks	Regulation of the gastrointestinal tract and prevention of cardiovascular disease and different forms of cancer, among other effects	[304]
*Lactobacillus casei* Shirota	Inulin, gelatin and whey protein	Probiotic delivery, stability in low-moisture matrices, and increase in shelf life	Cracker cookies	Preventing various health problems	[305]
Iron and ascorbic acid	k-Carrageenan or tapioca starch	Improves product stability by enhancing ascorbic acid retention and iron bioaccessibility under intestinal conditions	Refrigerated ready-to-eat pumpkin (*Cucurbita moschata* Duchesne ex Poiret)	Carrying micronutrients	[306]
Lytic bacteriophage	Chitosan	Antimicrobial delivery	Tomatoes	Enhanced microbial safety against *Escherichia coli* (minimizes foodborne pathogen risk through inactivation and growth inhibition)	[307]
Garlic essential oil	Chitosan	Increases antioxidant properties (synergistic effect), especially as a radical scavenger	Beef meatball	Antioxidant (anti-diabetic, anti-cancer, and anti-atherosclerotic activities)	[308]
Probiotic (*Lactobacillus rhamnosus* CECT 8361) and prebiotic (inulin and oligofructose)	Alginate	Synbiotic delivery, improved probiotic viability, antimicrobial activity, antilisterial effect	Blueberries	Probiotic carrier with antagonistic activity against fruit-borne pathogens and improvements regarding male fertility	[309]
Probiotic (*Bifidobacterium animalis* subsp. lactis BB-12) and prebiotic (inulin)	Whey protein isolate and alginate	Synbiotic delivery and improved probiotic strain viability throughout storage and throughout in vitro gastrointestinal digestion	Cereal bars	Antioxidant properties and supports gut health	[310]
Probiotic (*Lactobacillus plantarum* CECT 9567) and prebiotic (lactobionic acid)	Sodium alginate	Synbiotic delivery and improved probiotic strain viability after the simulated digestion	Cottage cheese	Improved health and/or reduces the risk of certain diseases	[257]
Prebiotics (oligofructose and inulin) and probiotic cultures (*Lactobacillus rhamnosus* and *Bifidobacterium animalis* subsp. *lactis*)	Alginate	Synbiotic delivery; increases the viability of probiotics; antimicrobial activity	Fresh-cut apple	Antioxidant activity; complementary strategy in the management of obesity	[300]
Probiotic strains (*Bifidobacterium lactis*, *Lactobacillus acidophilus*, and *Lactobacillus casei*)	Chitosan, sodium alginate and carboxymethyl cellulose	Antimicrobial preservation to extend shelf life and ensure stability	UF soft cheese	Digestive regulation, stimulating the immune system, lowering cholesterol levels, lactose intolerance, cancer prevention, and cardiovascular diseases	[297]
Tyrosol	Chitosan	Extends the shelf life and preserves the quality of fresh food	Tomatoes	Antioxidant, cardioprotective, antitumoral, anti-inflammatory, and neuroprotective properties	[263]
Probiotic bacteria (*Limosilactobacillus fermentum* NKN51 and *Lactobacillus brevis* NKN52)	Sodium caseinate and chia mucilage	Probiotic delivery, improving their viability and stability on bakery products	Wheat buns	Gut health support and disease risk reduction	[261]
Probiotic strain *Enterococcus faecium* FM11-2	Mucilage of cactus (*Opuntia ficus-indica*)	Probiotic delivery, improving the preservation and shelf life of the product	Fresh-cut apple slices	Antioxidant activity, potential gut health benefit from probiotic delivery	[311]
Probiotic (*L. acidophilus*) and prebiotic (agave fructans)	Sodium alginate	Synbiotic delivery, increases the viability of probiotics, improves thermal resistance and increases shelf life	Corn-based snack (churritos) almasalud	Reduction in the duration of diarrhea and reduction in body mass index in obese individuals	[312]
Seaweed (*Pelvetia canaliculate*)	Alginate and carrageenan	Reduces oil absorption, minimizes water loss, preserving fish succulence, and prevents fat oxidation during cooking	Mackerel (*Scomber scombrus*)	Reduction in the intake of saturated and trans fatty acids, preventing cardiovascular diseases	[313]
Encapsulated raspberry pomace powder	*Aloe ferox* gel	Increasing shelf life while minimizing weight loss and retaining firmness	Ready-to-eat pomegranate arils	High antioxidant, anti-mutagenic, and antihypertensive properties	[314]

In addition to the challenges of ensuring functionality under gastrointestinal conditions, consumer acceptance remains a critical factor. In this context, evaluating sensory attributes such as colour and appearance, odour, and taste is essential to determining the feasibility of incorporating functional ingredients into new food products. For instance, Alvarez et al. (2021) [300] reported that the addition of probiotic strains to a prebiotic–alginate coating negatively affected the product’s sensory quality at the end of storage. Apples containing *L. rhamnosus* showed browning, off-odours, and a deteriorated appearance after 8 days, with scores below the established acceptability limit. Furthermore, the complexity and variability of food matrices is another challenge to consider in these trials. For example, in the case of apple snacks [304], brushing dry and crunchy snacks with a liquid film-forming solution can soften them and impair their sensory properties. The problem of variability within batches, and even within individual fruits, is an accepted fact in these food matrices [309].

## 6. Overview on Social and Environmental Impact and Scalability

According to the United Nations’ 17 Sustainable Development Goals (SDGs) outlined in the 2030 Agenda, edible packaging can contribute to achieving some of them. In particular, Goal 2 (Zero Hunger) and Goal 3 (Good Health and Well-being) may be supported through the use of active edible materials [315]. These materials can extend food shelf life, enhance nutritional profiles by delivering bioactive compounds, and reduce food industry waste by utilizing it as a source of high-value ingredients. This integrated approach supports both food security and public health, while promoting more sustainable production systems.

Nonetheless, edible films and coatings must face a scalability challenge, since they are usually developed at lab scales and their acquisition is limited, leading to high production costs. Additionally, these biopolymers exhibit wide variability in molecular weight, chain branching, and purity in each batch, depending on their origin and extraction protocol [316]. Consequently, this inconsistency affects their brittleness, thermal stability, and barrier or mechanical properties, complicating reproducible processing.

Casting is the methodology most employed in coating/film formulation, and it is not compatible, as are other lab-scale methods, with industrial processes [317]. Many biopolymers cannot tolerate the thermal and shear stress of extrusion, resulting in film thickness variations or adhesion issues. Alternative technologies like electrospraying or electrospinning show lab-scale promise for active coating production, but their low yields and lack of uniformity make industrial upscaling difficult [317]. Other techniques, such as microbial biopolymer production (pullulan or xanthan gum), require specific environmental conditions, which increases operational costs and is vulnerable to contamination. The absence of standardized production protocols and the difficulties of processability and scalability lead to high production costs, challenging the commercializing of innovative packaging [318].

Furthermore, the use of biopolymers is eco-friendly, since their production exhibits lower non-renewable energy use, CO_2_ emissions, and overall greenhouse gas emissions, compared to conventional plastics and biobased alternatives (PHA, PLA, PBAT, etc.). Nonetheless, environmental concerns are raised when considering their life cycle assessments (LCAs) [319]. Many studies have focused on the most currently employed biobased and biodegradable materials, with little research found on the biopolymers reviewed in the present study [120,204]. Hence, considering bioplastics end of life, recycling (thermomechanical, chemical, and biological) may reduce greenhouse gas emissions, since the potential to replace virgin materials is found to be >80% [320,321]. However, the number of recycling cycles remains inconclusive [322]. On the other hand, landfilling and composting are among the most common disposal or degradation pathways, although none of them are inherently more environmentally favourable than energy recovery, nor the most employed [320,323]. Despite the fact that bioplastics can be landfilled without prior separation or cleaning, this method involves uncontrolled methane emissions, whose energy recoveries do not exceed 20%. Industrial composting was found by some authors to be the pathway with highest negative impact, due to high CO_2_ and N_2_O production or soil acidification, which negatively impact seed germination [319,324]. Conversely, anaerobic digestion has been found to be the degradation pathway with the lowest footprint, due to the use of biogas for electricity production [319].

Consequently, bioplastics also lead to environmental impacts, including eutrophication due to biomass cultivation, acidification, or land use change. Therefore, the scalability of the production of active and biodegradable polymers should be assessed in a way that aims to incorporate investigations into their real-world use. Although the production of bioplastics leads to lower energy costs and greenhouse gas emissions, more research and a better understanding of the LCA and end of life of bioplastics must be achieved in order to reduce their environmental impact by achieving a circular economy.

## 7. Safety and Regulation of Active and Edible Coatings

Edible coatings are subject to regulations that apply to food as they are part of the edible portion of the food product. Thus, the substances used in the preparation of edible coatings must be of food grade. In Europe the components used in the manufacture of edible coatings are mainly constituted by additives which are regulated under the Regulation (EC) No 1333/2008 of the European Parliament and of the Council on food additives [325] and they are included in the union’s list of food additives published in Commission Regulation (EU) No 1129/2011 [326]. Moreover, they must meet specifications concerning purity as stated in Commission Regulation (EU) No 231/2012 [327] and this must be indicated on the label either with the chemical name or the E number.

In the United States the substances used in edible coatings must be classified as Generally Recognized As Safe (GRAS). The Food and Drug Administration (FDA) provides an inventory of substances allowed in edible coatings, which fall into the categories “Food additives permitted for direct addition to food for human consumption” and “Secondary direct food additives permitted in food for human consumption”. In addition, other substances including copolymers (e.g., vinyl chloride–vinylidene chloride copolymer), dispersing adjuvants (e.g., polyethylene glycol), film formers (e.g., sodium lauryl sulphate), and adjuvants (e.g., polyvinylpyrrolidone) are also allowed [7,328,329,330].

Other important aspect that should be considered include the presence of ingredients that are potentially allergenic, which also should be declared in the label [7,328].

Active coatings which are not part of food and are not intended to be consumed together with the food are regulated by Commission Regulation (EC) No 450/2009 on active and intelligent materials and articles intended to come into contact with food. However, it is worth mentioning that active substances that are intentionally added with the purpose of being released into the food and are intended to have a technical effect on the food must meet the requirements of food additive legislation [331].

Regarding bioactive substances, currently plant extracts are being investigated to be incorporated into active coatings, due to, on the one hand, the better acceptance of natural compounds by the consumer and, on the other, the use of natural resources. However, a very limited number of plant extracts (e.g., extracts of rosemary) have been authorized as additives in the EU, which hinders the commercialization of these active systems. Therefore, the regulatory requirements remain a critical issue for their commercialization.

## 8. Conclusions and Future Perspectives

Studies on edible films and coatings are a trending topic in sustainable packaging that enhances food functionality and extends shelf life. Biobased materials from food by-products have emerged as a sustainable alternative, reducing environmental impact while contributing to circular economy strategies. Despite their potential, several challenges remain before these materials can achieve large-scale commercialisation. Their mechanical and barrier properties are still inferior to those of conventional plastics, and thus the incorporation of additives and the use of advanced techniques, such as EHDA or spraying for nano- or micro-structured multilayered coatings, should be further explored. Additionally, the incorporation of bioactive compounds and natural extracts not only improves food safety and quality but also enables the development of nutrient-enriched and functionalized foods. However, regarding the controlled release of pre- and probiotics, nutrients, and bioactives, key parameters such as compatibility, stability during processing and storage, bioactivity, and functionality require deeper investigation. Edible coatings and films are recognized as a safe, renewable, low-cost, and sustainable. However, barriers remain, including consumer acceptance, scalability, and the high costs associated with industrial production (e.g., controlled release nanosystems). Bio-based materials and encapsulation technologies need to become cost-competitive with conventional alternatives. For regulatory approval, compliance with food safety standards, and allergenicity assessment, digestibility and toxicity testing remain critical requirements. Therefore, future efforts should not only focus on technical progress but also address consumer education, regulatory support, the integration of circular economy principles, and cost competitiveness to facilitate broader adoption.

Importantly, this review goes beyond summarizing the general fundamentals of edible coatings and films by focusing on underexplored aspects that can drive future innovation in the field. Particular emphasis is placed on the integration of new biopolymer sources and nanostructured materials to enhance mechanical and barrier properties, the development of controlled release systems for bioactive compounds as an emerging but often overlooked functionality, and the application of edible coatings as carriers of nutrients and bioactives to support the design of functional foods. By consolidating recent advances in these areas and highlighting the existing knowledge gaps, this review provides a roadmap for researchers, industry, and regulators to accelerate the transition of edible coatings and films from experimental development to commercial reality, ultimately contributing to more sustainable and health-oriented food systems.

## Figures and Tables

**Table 1:** Examples of industrial commercially available edible coatings and films for food packaging applications from natural resources.

Manufacturer	Commercial Name–Patent Number	Specifications	Application
Bio2Coat SL/Universitat Politecnica de Catalunya (Spain) Website: https://bio2coat-group.com/ (accessed on 27 August 2025)	No trade name	Coating and film formulation: Mix of biopolymers, natural plasticizers, and bioburdens from food by-products and agrifood-sourced plant-based by-products (e.g., tomato concentrate for rice packaging; cocoa concentrate for packaged rice). Currently filing patents Effects: Maintains freshness and preserves firmness and humidity. Antimicrobial properties. Prolongs fruit shelf life by 40% by reducing fruit weight loss by 50%. Suitable for celiacs and allergen-free.	Fruits (spraying, nebulizing)
Poly-natural (Chile) Website: https://polynatural.com/ (accessed on 27 August 2025)	Shel-life^®^	Coating formulation: Plant-based coating. Organic oils and extracts from vegetables (not specified). Effects: Preserves firmness of cold-stored fruit. Reduces dehydration (20–30%). Maintains internal quality avoiding browning.	Fruits (spraying)
Notpla * (UK) Website: https://www.notpla.com/ (accessed on 27 August 2025)	Ooho Patent N: US20200047927A1 (Method of encapsulating liquid products)	Formulation: Algae-based films. Products: containers, trays, bags, sachets, rigid cutlery, energy gel pods, etc. High-performance barrier to fats and moisture (coating in containers). Effects: Generally intended to provide edible packaging and substitute plastics and PFAS, depending on final application.	Food products: oil, water… Other products: laundry, bath oil…
AgroSustain (Switzerland) Website: https://www.agrosustain.ch/ (accessed on 27 August 2025)	Afondo^®^ Patent N: WO 2024/110661 A1 (Edible coating for use as a plant biostimulant)	Coating formulation: o/w microemulsion made with diverse vegetable oils. Effects: Extends shelf life of crops, replaces post-harvest treatments, waste reduction up to 50%, taste preservation, reduces water loss up to 70%, minimizes plastic packaging use.	Fruits, vegetables, and flowers (spraying, dipping)
LiquidSeal HoldingBV (The Netherlands) Website: https://www.liquidseal.nl/ (accessed on 27 August 2025)	Liquidseal^®^ Patent N: WO 2020/226495 A1 (Edible coating composition for coating fresh harvest products)	Coating formulation: aqueous emulsion. Effects: Limits infections and cross-contamination. Improves taste and appearance, extends shelf life, maintains product quality, preserves firmness, and reduces weight loss.	Fruits: avocado, citrus, mango, and papaya. Vegetables: cucumbers. Flowers (spraying, dipping)
Mori (USA) Website: https://www.mori.com/ (accessed on 27 August 2025)	Mori^®^	Coating formulation: silk protein Effects: improves quality and extends freshness of food	Perishable food (wherever a food interacts with water) (dipping, spraying, glazing)
Margrey Industrial SA (Mexico)	No trade name Patent N: WO 2018/174699 A1 (Wax composition for coating fruit and vegetables)	Coating formulation: emulsion of carnauba wax, shellac, pine resin, etc. Effects: gives high shine, does not alter organoleptic properties, decreases weight loss, permeable to gas exchange, antifungal activity, improves shelf life.	Fruits and vegetables (fluidization, dipping, spraying or roller impregnation)
Kerry Group Services International Ltd. (Ireland) Website: https://www.kerry.com/ (accessed on 27 August 2025)	No trade name Patent N: US 2011/0014333 A1 (Oil-based coating for baked food products)	Coating formulation: emulsion of soybean salad oil, rosemary extract, oleoresin turmeric, paprika oleoresin, etc. Effects: reduces rancidity and increases shelf life.	Baked food products (spraying, dipping)
Tomorrow Machine and Eckes Granini (Sweeden) Website: https://goneshells.com/ (accessed on 27 August 2025)	GoneShells^®^	Formulation: Potato starch-based bottles. Currently a prototype Effects: water-resistant. Bio compostable. General packaging purposes.	Juices
Do eat (Belgium) Website: https://www.food.be/companies/do-eat (accessed on 27 August 2025)	No trade name	Formulation: Potato starch. Products: bags, vessels, etc. Effects: Gluten free and suitable for vegetarians. Neutral flavour. General packaging purposes.	Bakeries, take-away food (chips, bagels.). Convenience food, prepared meals and dishes
Valdís Steinars (Iceland)	Bioplastic Skin	Formulation: gelatine meat by-products. Effects: General packaging purposes. Transparency is pursued to assess freshness through visual indication.	Meat
Evoware * (Indonesia) Website: https://rethink-plastic.com/home/ (accessed on 27 August 2025)	No trade name	Formulation: seaweed, cassava starch, sugarcane bagasse, etc. Ingredients depend on the formulation. Products: bags, sachets, straws, food containers, cups, etc. Effects: General packaging purposes.	Food and beverages
Caragum (France) Website: https://www.caragum.com/en/ (accessed on 27 August 2025)	Fibrecoat^®^	Coating formulation: plant fibre and seaweed extract. Effects: Reduction in fat absorption in fried breaded products (26.4% reduction in fat content), improves crunchiness, slightly improves the organoleptic properties of fried breaded products	Fried breaded products (spraying)

* Not all the products developed by the company are edible.

## Data Availability

No new data were created or analyzed in this study.
